# Quantifying Pilot Operational Deviations Based on Flight Data via Inverse Reinforcement Learning and Conformal Prediction

**DOI:** 10.3390/s26196086

**Published:** 2026-09-25

**Authors:** Taoyuan Chen, Zhenxing Gao, Yangyang Zhang

**Affiliations:** College of General Aviation and Flight, Nanjing University of Aeronautics and Astronautics, Nanjing 211100, China; sz2420008@nuaa.edu.cn (T.C.); zhang_yy@nuaa.edu.cn (Y.Z.)

**Keywords:** flight data monitoring, operational deviations, GMM-HMM, inverse reinforcement learning, conformal prediction

## Abstract

Threshold-based flight data monitoring (FDM) identifies parameter exceedances but provides limited visibility into gradual operational deviation within nominal operating bounds. This study presents a three-layer framework for continuous operational deviation quantification from quick access recorder (QAR) data. A standard operating procedure (SOP)-constrained Gaussian mixture model–hidden Markov model (GMM-HMM) infers 27 procedural states from flight parameters, aligning latent state representation with operational procedure. From these state sequences, maximum entropy inverse reinforcement learning (IRL) recovers an expert value function that estimates execution quality at each time step. Split conformal prediction calibrates detection thresholds with distribution-free coverage guarantees, while counterfactual analysis traces identified deviations to their procedural origin. Learned from real flight data, the recovered reward function assigns 1.6 times greater weight to lateral and speed stability than to vertical stability. Split conformal calibration yields an empirical false alarm rate of 4.2% under a nominal 5% significance level. Counterfactual analysis identifies vertical speed management as the dominant source of procedural deviation. These results support the proposed framework as a candidate continuous safety performance indicator that surfaces gradual operational deviation that threshold-based exceedance monitoring would not flag.

## 1. Introduction

Flight data monitoring (FDM) programs underpin proactive civil aviation safety management, leveraging quick access recorder (QAR) data to systematically evaluate pilot operational quality and aircraft system performance [1,2,3]. This need is especially acute during final approach and landing, which account for only 4% of flight duration but contribute to 48% of fatal aviation accidents [1]. Authoritative guidance on stable approaches and on the use of flight data for safety performance monitoring is distributed across International Civil Aviation Organization (ICAO) safety management documentation and national advisory material on flight operational quality assurance [2,4,5]; these sources frame the operational objective that the framework in this paper targets.

Historically, FDM has evaluated safety through threshold-based exceedance detection. While effective for identifying categorical safety violations, this paradigm cannot quantify subtle variations in procedural compliance that occur entirely within nominal operating limits. Quantifying such variations is fundamentally difficult because procedural quality has no ground-truth label and deviations are distributed across multiple interacting parameters. A pilot who maintains a stable descent at −700 ft/min and one who oscillates between −400 and −1000 ft/min both receive zero FDM alarms, yet the procedural execution quality differs substantially. In ICAO safety management systems (SMS) terminology [4,5], current FDM exceedance counts provide limited visibility into operational deviation before exceedance events occur. Operational FDM practice is broader than simple exceedance counting and now includes trend monitoring, precursor identification, and risk-weighted event analysis within airline safety management systems [2,3]; even these more advanced treatments remain event-oriented, however, because they characterize discrete alarms or precursors rather than a continuously varying measure of procedural execution quality. Operational safety management would benefit from a continuous leading indicator that tracks the trajectory of procedural execution quality before any single parameter reaches an exceedance boundary. More broadly, the aviation industry is increasingly exploring predictive, data-driven approaches to complement reactive threshold monitoring for aircraft health management [6].

Unsupervised deep learning has been widely applied to detect anomalies in multidimensional QAR time-series data. These approaches span attention-enhanced variational autoencoders [7], hybrid autoencoder architectures [8], multiple-instance learning for precursor identification [3], dual-branch architectures for imbalanced flight data [9], and comparative studies across LSTM, GRU, CNN, and Transformer models [10]. General-purpose time-series anomaly detectors, including the Anomaly Transformer [11] and LSTM-based sensor anomaly detection across multiple operational states [12], and LSTM-GPT2 fusion networks for multivariate industrial sensor time series [13], have also been adapted to flight data monitoring. Existing approaches treat flight monitoring as an anomaly classification task [14,15] and produce scalar anomaly scores. These scores indicate that something is unusual but do not identify which procedural element deviated or what corrective action to take—capabilities that a continuous safety performance indicator (SPI) requires. Beyond black-box anomaly detection, Hidden Markov Models have been applied to flight data for phase recognition [16]; our work inherits this probabilistic modeling paradigm but operates at finer granularity, modeling 27 discrete procedural states within a single approach phase. Behavior-modeling methods, by contrast, treat aircraft piloting as a sequential decision-making process governed by physical constraints and standardized operating procedures [17]. Operational deviation is closely related to pilot workload and human performance, and machine-learning methods have shown promising results in assessing pilot workload from physiological signals during both simulated and real flight, complementing flight-data-based monitoring with a direct measure of the human operator state [18,19].

Inverse reinforcement learning (IRL) infers reward functions from expert demonstrations [20,21,22]. It has recently been applied to pilot behavior modeling for both manned and unmanned aircraft [23,24], human-in-the-loop behavior modeling [25], landing procedure optimization [26], and wind shear escape training [27]. Compared with generative adversarial imitation learning [28], which learns a policy without an explicit reward representation, IRL yields a transparent decision function: the learned reward directly encodes which actions are preferred and by how much, expressed as a linear combination of operational features. In safety-critical aviation domains, this transparency is particularly valuable for flight crew debriefing and operational guidance, where post hoc explanations for black-box models are often insufficient for high-stakes decision-making [29,30,31]. The learned reward function provides a quantifiable, interpretable score of operational quality for each timestep. However, this score alone provides no statistical guarantee on the false alarm rate at any threshold, limiting its operational utility for alerting decisions.

Conformal prediction (CP) [32,33] provides distribution-free finite-sample statistical guarantees. Traditional parametric threshold-setting methods common in FDM practice, such as the three-sigma rule, assume normally distributed scores. This assumption may not hold for flight data, where score distributions can be skewed, heavy-tailed, or multi-modal. CP requires no distributional assumptions, guaranteeing false alarm rate control for any underlying score distribution. Anomaly-detection pipelines in practice usually select a decision threshold heuristically, for example, on a held-out subset or by visual inspection of a score distribution [15]; such thresholds carry no finite-sample statement about the resulting false alarm rate. Split conformal prediction differs in that the threshold is derived from a calibration set and carries an explicit guarantee at a chosen error level. CP has been applied to time-series forecasting [34,35], dynamic trajectory planning [36], aircraft navigation during landing [37], and flight test safety monitoring [38]. Here, we integrate CP-based calibration with IRL deviation metrics to quantify operational deviations with distribution-free statistical guarantees, and combine both with counterfactual explanations for procedural deviation diagnosis.

Against this background, this study addresses three research questions. The first is whether the quality of procedural execution during approach can be quantified continuously from QAR data without anomaly labels, so that gradual deviations occurring within nominal operating limits become visible. The second is whether maximum-entropy IRL recovers an expert reward function whose valuation of the 27 procedural states is interpretable, stable under alternative expert definitions, and consistent with independent physical signals. The third is whether split conformal prediction can bound the false alarm rate of such a continuous quality score while counterfactual analysis identifies which procedural element a detected deviation involves.

This paper presents a three-layer framework for continuous operational deviation quantification based on flight data. The framework integrates procedurally grounded state inference, expert value learning, and statistical calibration into a single pipeline. An SOP-constrained GMM-HMM maps flight parameters to 27 procedurally defined latent states, grounding state inference in operational procedure. From these state sequences, MaxEnt IRL recovers a transparent expert value function whose linear feature weights, inspectable by domain experts, score execution quality at each timestep. Split conformal prediction then calibrates these scores with distribution-free false alarm rate guarantees requiring only a held-out set of normal flights, while counterfactual analysis traces identified deviations to their procedural origin. Relative to our earlier work, which introduced the SOP-constrained GMM-HMM state-inference backbone, the contribution of this paper is the integration, above that backbone, of three mutually consistent layers: maximum-entropy IRL for recovering an expert reward function, split conformal prediction for distribution-free false alarm control, and SOP-constrained counterfactual analysis for procedural diagnosis. Sharing a single MDP value function, these layers jointly convert a state-inference model into a continuous, explainable, and statistically bounded indicator of operational deviation. The framework is formulated in methodologically general terms so that its definition does not depend on a specific aircraft type, route, or flight phase, and it requires no anomaly labels for training or calibration. It is evaluated on approach-flight data that span two Airbus aircraft families, the A319 and A330, and multiple operators on a single high-plateau route through systematic ablation, cross-definition expert selection, sensitivity analysis, and comparison with multiple baseline methods; this experimental scenario serves as one validation instance rather than establishing the framework’s empirical scope.

## 2. Methodology

The proposed framework follows a three-layer architecture, illustrated in Figure 1. The Measurement Layer performs state inference via an SOP-constrained GMM-HMM that maps flight parameters to 27 procedurally defined latent states. The Reference Layer learns an expert value function through MaxEnt IRL and derives a per-timestep Q-value compliance gap. The Assurance Layer provides statistical guarantees through split conformal calibration and traces identified deviations to their procedural origin via counterfactual analysis. The Per-Flight Output consolidates these into a compliance score, a conformal *p*-value, and corrective action guidance.

### 2.1. Problem Formulation and SOP State Encoding

Let $F=\{F_{1},F_{2},\ldots ,F_{N}\}$ be a set of *N* flights. Each flight $F_{i}$ during the approach phase is represented as a sequence of $T_{i}$ observations $O^{\left(i\right)}=\left[o_{1},o_{2},\ldots ,o_{T_{i}}\right]$, where each observation $o_{t}\in R^{9}$ consists of nine flight parameters: indicated airspeed (IAS), pitch angle, roll angle, engine fan speed (N1), localizer deviation (LOC), glideslope deviation (GLIDE), vertical speed ($V_{\mathrm{SPD}}$), longitudinal acceleration (ACC), and heading rate ($H_{\mathrm{RATE}}$). Each observation is associated with a hidden standard operating procedure (SOP) state $s_{t}\in S=\left\{0,1,\ldots ,26\right\}$ representing a discrete combination of three flight control axes.

These nine parameters are drawn from the full QAR parameter suite through selection guided by domain knowledge rather than statistical screening. Three parameters serve as the state-defining control variables: vertical speed for descent rate management, heading rate for turn execution, and longitudinal acceleration for energy management. These are the parameters against which SOP compliance is assessed. The remaining six (IAS, pitch, roll, N1, LOC, and GLIDE) provide complementary aircraft-state and flight-path-deviation context, enriching the GMM emission models with physical observables that correlate with procedural execution quality without entering the state definition. This nine-parameter set constitutes the standard flight operational quality assurance (FOQA) monitoring parameter suite for the approach phase [2] and achieves operational completeness across all three control axes without requiring statistical dimensionality reduction techniques that would sacrifice the interpretability of individual parameters.

The 27 hidden states are defined by the Cartesian product of three flight-mode dimensions, each discretized into three operationally meaningful categories. The category boundaries are calibrated through a joint procedure: the stabilized approach criteria from the flight crew operating manuals (FCOMs) of the Airbus A319/A330 series aircraft operating this route define the operational reference points of stable descent at approximately −700 ft/min on a 3° glideslope, heading hold, and speed hold; the specific numerical thresholds are then refined against the empirical distribution of 3325 QAR flights to ensure each category captures a distinct flight regime. This approach, informed by the FCOM and calibrated with data, ensures that the discretization is both operationally grounded and empirically justified. The division between prescription and data tuning is therefore explicit: the FCOM supplies the operational reference points, while the category boundaries are engineering parameters refined against the empirical data distribution. The stable-descent vertical band from −1000 to −400 ft/min is intentionally wide because the sink rate of a stabilized approach varies with aircraft mass, wind, and energy state; on the high-plateau route considered here, the A319 and A330 families operate at comparable reference speeds, so a single shared band avoids fragmenting a continuous descent profile into spurious transitions, and the −1000 ft/min boundary marks the onset of sink rates that stabilized-approach guidance treats as unstable. The three dimensions are:

- Vertical (*v*): 0 = sink-fast ($V_{\mathrm{SPD}}<-1000$ ft/min), 1 = stable descent ($-1000\leq V_{\mathrm{SPD}}\leq -400$ ft/min), 2 = sink-slow ($V_{\mathrm{SPD}}>-400$ ft/min)
- Lateral (*l*): 0 = left-turn (heading rate $<{-1.0}^{\circ }$/s), 1 = heading-hold ($\left|\mathrm{heading}\;\mathrm{rate}\right|\leq 1.0^{\circ }$/s), 2 = right-turn (heading rate $>1.0^{\circ }$/s)
- Speed ($s_{p}$): 0 = decelerate (ACC <−2.0 knots/s), 1 = speed-hold (|ACC|≤2.0 knots/s), 2 = accelerate (ACC >2.0 knots/s)

The state index is computed as $s=v\times 9+l\times 3+s_{p}$. State 13 (v=1,l=1, $s_{p}=1$) represents the nominal SOP-compliant configuration: stable descent, heading hold, speed hold.

The 27-state discretization is a modeling construct: the specific threshold values are engineering choices calibrated to the aircraft’s approach characteristics rather than unique FCOM prescriptions. The validity of this encoding is established empirically through two independent procedures: (i) an ablation study showing that replacing the SOP-constrained transition matrix with a uniform prior degrades state inference, demonstrating that the discretization captures meaningful procedural structure; and (ii) a robustness check showing that an entirely SOP-agnostic expert selection criterion recovers a reward function highly correlated with the SOP-informed one, indicating that the learned quality structure is robust to threshold choices.

### 2.2. SOP-Informed GMM-HMM for State Inference

The GMM-HMM, shown in Figure 2, serves as the probabilistic state inference backbone of the framework. It is a standard statistical model [39]; the SOP-informed 27-state discretization and SOP-constrained transition matrix were introduced in our prior work and are summarized here for completeness. The model is defined by λ=(π,A,Θ), where $\pi \in R^{27}$ is the initial state distribution, $A\in R^{27\times 27}$ is the state transition probability matrix, and $\Theta ={\left\{\theta_{s}\right\}}_{s=0}^{26}$ are the emission model parameters. Each emission model $\theta_{s}={\left\{w_{s,k},\mu_{s,k},\Sigma_{s,k}\right\}}_{k=1}^{K}$ is a *K*-component diagonal-covariance GMM. The emission probability of observation $o_{t}$ given state $s_{t}=j$ is

(1) $$b_{j}\left(o_{t}\right)\equiv P\left(o_{t}|s_{t}=j;\theta_{j}\right)=\sum_{k=1}^{K}w_{j,k}\;N\;\left(o_{t}\;|\;\mu_{j,k},\mathrm{diag}\;\left(\sigma_{j,k}^{2}\right)\right)$$

where $w_{j,k}\geq 0$ are the mixing weights with $\sum_{k=1}^{K}w_{j,k}=1$ for each state *j*, N(·∣μ,Σ) denotes the multivariate Gaussian density, and $\mathrm{diag}(\sigma_{j,k}^{2})$ is the diagonal covariance matrix parameterized by the per-dimension variance vector $\sigma_{j,k}^{2}\in R_{>0}^{9}$.

#### 2.2.1. SOP Transition Constraint

The defining structural constraint is on the transition matrix A:

(2) $$A_{i,j}>0\Leftrightarrow \mathrm{is}\text{\_}\mathrm{sop}\text{\_}\mathrm{compliant}\left(i,j\right)$$

where a transition is SOP-compliant if and only if it changes at most one dimension by at most one category:

(3) $$\mathrm{is}\text{\_}\mathrm{sop}\text{\_}\mathrm{compliant}\left(i,j\right)=\left(\sum_{d}I\left[c_{d}\left(i\right)\neq c_{d}\left(j\right)\right]\leq 1\right)\wedge \left(\max_{d}\left|c_{d}\left(i\right)-c_{d}\left(j\right)\right|\leq 1\right)$$

where I[·] is the indicator function and $c_{d}\left(s\right)\in \left\{0,1,2\right\}$ is the category of state *s* in dimension $d\in \{v,l,s_{p}\}$. Of the $27^{2}=729$ possible transitions, only 135 (18.5%) are SOP-compliant. This constraint formalizes the modeling assumption that pilots predominantly execute single-axis, incremental corrections during stabilized approach and serves as a strong structural prior that improves state inference in the presence of heavy class imbalance.

#### 2.2.2. Training and Inference

The model was trained via supervised initialization followed by maximum likelihood estimation. SOP state labels were first assigned to each training timestep by rule-based thresholding of the nine flight parameters. The transition matrix A and initial distribution π were then estimated via maximum likelihood from the labeled state sequences, with $A_{i,j}$ forced to zero for all SOP-invalid transitions (Equation (3)). Finally, for each state *s*, a *K*-component GMM with diagonal covariance was fit to the observations assigned to that state, with K=2 chosen as the balanced trade-off between aggregate inference accuracy and minority-state detection; K=1 yields slightly higher overall weighted F1 but degrades per-state F1 for minority states S12 and S14. Diagonal covariance was chosen over full covariance for computational efficiency; the variance floor of $10^{-4}$ serves as implicit regularization that achieves comparable numerical stability to full-covariance regularization at lower cost [39]. The variance floor also prevents degenerate covariances from states with limited observations.

Given a trained model λ and an observation sequence O, the joint probability of a state sequence $s=(s_{1},\ldots ,s_{T})$ and the observation sequence factorizes as

(4) $$P\left(s,O|\lambda \right)=\pi_{s_{1}}\prod_{t=2}^{T}A_{s_{t-1},s_{t}}\prod_{t=1}^{T}b_{s_{t}}\left(o_{t}\right)$$

where $b_{s_{t}}\left(\cdot \right)$ is the GMM emission density defined in Equation (1) and $\pi_{s_{1}}$ is the initial state probability. The most likely state sequence is inferred via Viterbi decoding:

(5) $$\hat{s}=\arg\max_{s}P\left(s|O;\lambda \right)$$

We adopt Viterbi decoding for state inference because the downstream compliance scores operate on discrete state sequences; the Viterbi path provides the single most probable joint state sequence under the SOP-constrained model.

The HMM operates on the same 27 SOP-defined states as the rule-based encoding. Its contribution is three capabilities that threshold rules cannot provide: soft state posterior probabilities $P(s_{t}|o_{t})$ rather than hard binary assignments; a principled model of state transition probabilities between SOP states, which the rules have no concept of; and a probabilistic framework that supports the downstream MDP formulation and Q-value compliance gap computation. The state labels used for supervised initialization are the SOP definitions themselves; the HMM’s value lies in modeling the probabilistic structure of how pilots move through these states.

#### 2.2.3. Physics-Informed Emission Regularization

While the SOP constraint on the transition matrix provides a strong structural prior, the Gaussian mixture model (GMM) emission components are learned purely from data without physical knowledge. We introduce an emission regularization step informed by physics, which applies soft physics constraints to GMM emission parameters after expectation-maximization (EM) training. The regularization strength is controlled by $\lambda_{\mathrm{phys}}$.

Three physics constraints are implemented. In the following, superscripts on emission means use zero-based indexing to reference specific flight parameters: [0] = IAS, [1] = pitch, [2] = roll (roll angle), [3] = N1, [4] = LOC (localizer deviation), [5] = GLIDE (glideslope deviation), [6] = $V_{\mathrm{SPD}}$ (vertical speed), [7] = ACC (longitudinal acceleration), [8] = $H_{\mathrm{RATE}}$ (heading rate). $\mu_{k}^{\left[2\right]}$ denotes the mean of the roll angle dimension for component *k*.

C1: Coordinated Flight. In a coordinated turn, roll angle and heading rate share the same sign. For each GMM component *k*, if $\mathrm{sign}\left(\mu_{k}^{\left[2\right]}\right)\neq \mathrm{sign}\left(\mu_{k}^{\left[8\right]}\right)$:

(6) $$\mu_{k}^{\left[8\right]}\leftarrow \left(1-\lambda_{\mathrm{phys}}\right)\cdot \mu_{k}^{\left[8\right]}+\lambda_{\mathrm{phys}}\cdot \mathrm{sign}\left(\mu_{k}^{\left[2\right]}\right)\cdot \left|\mu_{k}^{\left[8\right]}\right|$$

C2: ILS (instrument landing system) Coherence. Pilots track localizer and glideslope beams simultaneously, so LOC and GLIDE deviation magnitudes should be correlated. When $|\mu_{k}^{\left[4\right]}-\mu_{k}^{\left[5\right]}|>0.02$:

(7) $$\mu_{k}^{\left[i\right]}\leftarrow \left(1-\lambda_{\mathrm{phys}}\right)\cdot \mu_{k}^{\left[i\right]}+\lambda_{\mathrm{phys}}\cdot \mathrm{sign}\left(\mu_{k}^{\left[i\right]}\right)\cdot \bar{m},\;i\in \left\{4,5\right\}$$

where $\bar{m}=\left(\right|\mu_{k}^{\left[4\right]}\left|+\right|\mu_{k}^{\left[5\right]}\left|\right)/2$.

C3: Descent-Acceleration Anti-correlation. During approach, pilots reduce thrust when descending. When $V_{\mathrm{SPD}}<-500$ ft/min and ACC >0.5 knots/s:

(8) $$\mu_{k}^{\left[7\right]}\leftarrow \left(1-\lambda_{\mathrm{phys}}\right)\cdot \mu_{k}^{\left[7\right]},\;\mathrm{if}\;V_{\mathrm{SPD}}<-500\;\mathrm{ft}/\min\;\mathrm{and}\;ACC>0.5\;\mathrm{knots}/s$$

These constraints are applied post-EM as a soft projection and do not participate in the likelihood optimization, ensuring compatibility with any GMM-HMM training pipeline. The regularization strength $\lambda_{\mathrm{phys}}=0.05$ is found to provide the optimal trade-off.

The GMM-HMM identifies where the pilot is in SOP state space at each timestep. It does not, however, answer how well the pilot is flying. State occupancy alone cannot distinguish a skilled pilot who reaches state 13 efficiently from one who oscillates through non-nominal states before eventually stabilizing. To quantify this distinction, we need a quality measure over states and transitions: a reward function. Standard operating procedures are prescriptive: they define which transitions are permissible but not which are preferable. Within the 135 SOP-compliant transitions, some are clearly more desirable than others, such as moving toward state 13 versus moving away from it. Manually specifying a reward function to capture these preferences would require committing to a particular weighting of operational factors—precisely the judgment that we aim to learn from data. We therefore formulate SOP compliance evaluation as an inverse reinforcement learning problem: given expert pilot demonstrations, can a reward function be learned that assigns higher value to states and transitions characterizing skilled flight?

### 2.3. Maximum Entropy IRL for Reference Policy Learning

We formulated SOP compliance evaluation as a Markov decision process (MDP) and applied maximum entropy inverse reinforcement learning (MaxEnt IRL) [22] to learn the reward function from expert demonstrations, eliminating the need for manually defined scoring heuristics. Figure 3 illustrates the IRL pipeline.

#### 2.3.1. MDP Formulation over SOP States

We define an MDP M=(S,A,T,R,γ) over the 27 SOP states:

- States S={0,1,…,26}: the 27 SOP states.
- Actions: from state *i*, the admissible actions are A(i)={j:sop_ok(i,j)}, where sop_ok is the SOP constraint defined in Equation (3).
- Transitions T: deterministic; selecting action *j* from state *i* transitions to state *j* with probability 1. A deterministic model is adopted because the SOP transition constraint, Equation (3), already restricts the action space to single-axis, single-category corrections; within this constrained space, the state outcome of each action is uniquely determined. The stochastic extension is discussed later.
- Reward R(s): a state-based reward function. We adopt R(s) rather than R(s,a) because, in SOP evaluation, what matters is being in a compliant state rather than the specific action taken to arrive there. State-only rewards also simplify identifiability: under entropy-regularized IRL, state-action rewards are identifiable only up to potential-based shaping transformations, whereas state-only rewards reduce this ambiguity [40]. Recent theoretical work has characterized the nonuniqueness of IRL solutions and established convergence guarantees for observer-based estimation [41].
- Discount γ=0.95, which yields an effective planning horizon of 20 steps (1/(1−γ)), corresponding to 20 seconds at the 1 Hz sampling rate. This horizon captures approach-phase decision sequences while remaining consistent with standard MaxEnt IRL practice.

The MaxEnt IRL principle posits that the probability of a trajectory $\tau =(s_{1},s_{2},\ldots ,s_{T})$ under reward weights θ is proportional to the exponential of its cumulative reward:

(9) $$P\left(\tau |\theta \right)=\frac{\exp\;\left(R(\tau ;\theta )\right)}{Z(\theta )}=\frac{\exp\;\left(\theta^{⊤}\phi \left(\tau \right)\right)}{Z(\theta )}$$

where $R\left(\tau ;\theta \right)=\sum_{t=1}^{T}R\left(s_{t};\theta \right)$ is the cumulative trajectory reward, $\phi \left(\tau \right)=\sum_{t=1}^{T}\phi \left(s_{t}\right)$ is the trajectory feature count, and $Z\left(\theta \right)=\sum_{\tau^{\prime }}\exp\left(\theta^{⊤}\phi \left(\tau^{\prime }\right)\right)$ is the partition function summing over all feasible trajectories. Equation (9) is the unique distribution that maximizes trajectory entropy subject to the constraint that expected feature counts match those of the expert demonstrations, giving the framework its “maximum entropy” characterization.

Under this distribution, the optimal policy satisfies

(10) $$\pi \left(a=j|s=i\right)\propto \exp\left(R\left(j\right)+\gamma \cdot V^{*}\left(j\right)\right)$$

where the soft value function is

(11) $$V^{*}\left(i\right)=\log\sum_{j\in A(i)}\exp\left(R\left(j\right)+\gamma \cdot V^{*}\left(j\right)\right)$$

The reward function is parameterized as a linear combination of state features: $R\left(s;\theta \right)=\theta^{⊤}\phi \left(s\right)$. We designed ten interpretable features. Nine are binary indicators encoding the dimension-level category of each state: three per axis, covering vertical stability (stable, sink-fast, sink-slow), lateral (hold, left-turn, right-turn), and speed (hold, decelerate, accelerate). One is an indicator for state 13, the nominal SOP configuration where all three axes are in their middle categories. This minimal feature set ensures that every SOP state receives a unique feature signature while maintaining a compact, auditable parameterization. We adopted a linear reward structure because, in safety-critical domains, each learned weight must carry a direct operational interpretation that domain experts can independently validate; the trade-off between representational capacity and auditability favors the latter [40].

#### 2.3.2. MaxEnt IRL Optimization

Expert flights were identified as the top-20% of training flights ranked by the fraction of timesteps in state 13. Let $p_{13}$ denote this fraction for a given flight. In our dataset, this yields 541 expert flights with mean $p_{13}=95.4\%$ and 2119 non-expert flights with mean $p_{13}=92.6\%$.

The MaxEnt IRL objective is to find reward weights θ such that expected feature counts under the MaxEnt policy match empirical feature counts of expert demonstrations:

(12) $$\theta^{*}=\arg\max_{\theta }\;L\left(\theta \right)=E_{\mathrm{expert}}{\left[\phi \left(s\right)\right]}^{⊤}\theta -\log Z\left(\theta \right)$$

The gradient takes the intuitive form of matching feature expectations:

(13) $$\nabla_{\theta }L=E_{\mathrm{expert}}\left[\phi \left(s\right)\right]-E_{\pi_{\theta }}\left[\phi \left(s\right)\right]$$

We optimized via gradient ascent with learning rate η=0.08 for 200 iterations. At each iteration, the model expected feature count is computed from the steady-state visitation distribution under the MaxEnt policy:

(14) $$D_{\pi_{\theta }}\left(s\right)=E_{\pi_{\theta }}\;\left[\sum_{t=1}^{T}I\left[s_{t}=s\right]\right],\;E_{\pi_{\theta }}\left[\phi \left(s\right)\right]=\sum_{s\in S}D_{\pi_{\theta }}\left(s\right)\;\phi \left(s\right)$$

where $D_{\pi_{\theta }}\left(s\right)$ is the expected number of visits to state *s* over a trajectory of length *T*, estimated by rolling the MaxEnt policy forward (Equation (10)) from a uniform initial state distribution for a horizon of 200 steps.

#### 2.3.3. Q-Value Compliance Gap

Under the reward function learned by IRL, the state-action Q-function is defined as the immediate reward plus the discounted soft value of the successor state:

(15) $$Q^{*}\left(i,j\right)=R\left(j\right)+\gamma \cdot V^{*}\left(j\right)$$

where $V^{*}\left(j\right)$ is the soft value function defined in Equation (11). For a transition from state *i* to state *j*, the compliance gap is

(16) $$\mathrm{gap}\left(i,j\right)=\frac{Q^{*}\left(i,j^{*}\right)-Q^{*}\left(i,j\right)}{\max_{k\in A(i)}Q^{*}\left(i,k\right)-\min_{k\in A(i)}Q^{*}\left(i,k\right)}$$

where $j^{*}=\arg\max_{j\in A(i)}Q^{*}\left(i,j\right)$ is the optimal next state. When $\max_{k}Q^{*}\left(i,k\right)=\min_{k}Q^{*}\left(i,k\right)$, all admissible actions from state *i* have identical Q-values and gap(i,j) is defined as 0 (no preference among actions). In practice, this edge case occurs only for states with a single admissible action or with an uninformative reward, neither of which arises under the reward function learned by IRL. For a flight with state sequence ${\left\{s_{t}\right\}}_{t=1}^{T}$, the flight-level compliance score combines the mean value and compliance gap:

(17) $$C\left(F\right)=\beta \cdot \frac{1}{T}\sum_{t}{\hat{V}}^{*}\left(s_{t}\right)+\left(1-\beta \right)\cdot \left(1-\frac{1}{T-1}\sum_{t}\mathrm{gap}\left(s_{t},s_{t+1}\right)\right)$$

where ${\hat{V}}^{*}\left(s\right)=V^{*}\left(s\right)/\max_{s^{\prime }}V^{*}\left(s^{\prime }\right)$ is the value normalized to [0,1], and β=0.5.

### 2.4. Conformal Calibration and Counterfactual Explanation

To provide statistical rigor to flight-level compliance scoring, we employed split conformal prediction. It requires no assumptions about the score distribution and provides a finite-sample guarantee on the false alarm rate. Figure 4 illustrates the conformal workflow.

#### 2.4.1. Nonconformity Score

We define the nonconformity score for a flight *F* as the mean negative log-likelihood (NLL) of hidden Markov model (HMM) state transitions:

(18) $$S_{\mathrm{nc}}\left(F\right)=\frac{1}{T-1}\sum_{t=2}^{T}-\log A_{{\hat{s}}_{t-1},{\hat{s}}_{t}}$$

where ${\hat{s}}_{t}$ is the state decoded by the Viterbi algorithm at time *t*. Even among fully SOP-compliant flights, natural score variation exists due to differences in operational conditions and piloting style. The calibration set captures this variation by establishing the empirical distribution of nonconformity scores under normal operations, against which new flights are evaluated.

#### 2.4.2. Calibration and Inference

Of the 2660 model development flights, 2128 (80%) are randomly allocated to HMM and IRL training, and the remaining 532 (20%) are reserved exclusively for conformal calibration. The calibration, model training, and held-out test sets are mutually disjoint. For a new test flight $F^{*}$ with score $s^{*}=S_{\mathrm{nc}}\left(F^{*}\right)$, the conformal *p*-value is

(19) $$p\left(s^{*}\right)=\frac{1+|\{c\in C:c\geq s^{*}\}|}{n_{\mathrm{cal}}+1}$$

At significance level α, a flight is flagged as potentially non-compliant if $p(s^{*})<\alpha$. By the split conformal guarantee, for any test flight $F_{\mathrm{test}}$ drawn exchangeably with the calibration set [33]:

(20) $$P_{(F_{\mathrm{test}},C)∼D}\;\left(p\;\left(S_{\mathrm{nc}}\left(F_{\mathrm{test}}\right)\right)<\alpha \right)\leq \alpha$$

where the probability is taken over the joint draw of the calibration set C and the test flight from the same underlying distribution D. For α=0.05, this guarantees that at most 5% of truly SOP-compliant flights are falsely flagged, providing the distribution-free statistical assurance required for operational deployment.

We select the transition NLL as the nonconformity score rather than the IRL-based compliance gap because the transition NLL is independent of the expert selection heuristic used in IRL training, making the statistical guarantee self-contained and not contingent on any particular expert definition. The split conformal framework is agnostic to the choice of nonconformity score: the same calibration procedure applies identically to any flight-level score, including the Q-value compliance gap, provided higher scores correspond to flights more likely to be non-compliant. In operational practice, an airline may calibrate compliance thresholds directly on the IRL-based score using the identical procedure after verifying exchangeability of the calibration and test distributions.

#### 2.4.3. Counterfactual Explanation via SOP-Constrained Decoding

The counterfactual state sequence $s^{\mathrm{cf}}$ is generated via greedy forward decoding constrained by SOP-reachable states:

(21) $$s_{t}^{\mathrm{cf}}=\arg\max_{s\in R(s_{t-1}^{\mathrm{cf}})}P\left(s|o_{t}\right),\;t\geq 1$$

where $R\left(s_{t-1}^{\mathrm{cf}}\right)=\left\{j:\mathrm{is}\text{\_}\mathrm{sop}\text{\_}\mathrm{compliant}\left(s_{t-1}^{\mathrm{cf}},j\right)\right\}$. The per-step deviation cost quantifies the posterior probability sacrifice:

(22) $${\cos t}_{t}=\max_{s}P\left(s|o_{t}\right)-P\left(s_{t}^{\mathrm{cf}}|o_{t}\right)$$

A deviation is identified at timestep *t* when the state decoded by the Viterbi algorithm differs from the counterfactual: $s_{t}^{\mathrm{actual}}\neq s_{t}^{\mathrm{cf}}$.

The greedy decoding strategy is chosen over alternatives such as beam search for two reasons. First, the SOP constraint (Equation (3)) restricts the action space to at most nine single-axis corrections per state, so the branching factor is small and greedy search rarely misses the optimal path. Second, the counterfactual serves an explanatory rather than generative purpose: its role is to identify which specific timesteps and state transitions diverged from SOP-optimal behavior, not to produce a globally optimal trajectory. The counterfactual decoding (Equation (21)) uses HMM posterior probabilities $P(s|o_{t})$ for greedy state reconstruction, while the compliance gap (Equation (16)) is computed from the MDP Q-function. Both are constrained by the same SOP transition rules (Equation (3)), ensuring that the counterfactual trajectory is always SOP-valid with respect to the compliance scoring framework. This shared constraint makes the explanation and the score mutually consistent by construction, a property that is not guaranteed when explanation and scoring models are trained independently. The counterfactual represents the model’s learned notion of optimal behavior under the learned reward function. Its operational utility remains to be validated through pilot-in-the-loop evaluation.

## 3. Results

### 3.1. State Inference and IRL Reward Learning

To validate the proposed framework, we employ 3325 approach flights provided by the flight quality monitoring base of the China Academy of Civil Aviation Science and Technology, which aggregates operational data from multiple Chinese commercial operators without attributing individual flights to a single airline. All flights operate from ZULS in Lhasa to ZUUU in Chengdu, a high-altitude route where atmospheric turbulence has been characterized through eddy dissipation rate estimation [42]; because of the performance requirements of this high-plateau route, the operating fleet comprises Airbus A319 and A330 series aircraft. The evaluation therefore spans two aircraft families and multiple operators within a single-route environment, and Discussion Section 4.3 characterizes the scope of the resulting transferability claim. Flight data are extracted from quick access recorders at 1 Hz sampling rate. The approach phase is isolated by altitude gating from top of descent (typically FL120) to touchdown, yielding variable-length sequences (mean ≈ 135 s, range 113–165 s) of 9-dimensional feature vectors. The dataset is partitioned into a model development set (2660 flights, 80%) and a held-out test set (665 flights, 20%). All flights completed safely with zero FDM hard-threshold exceedances. Each flight record is preprocessed through a standardized pipeline. First, the approach phase is isolated via pressure altitude: data below FL120 and above 50 ft AGL are retained, and the final 5 s before touchdown are excluded to avoid ground-effect transients. Second, missing values, which affect <0.3% of samples and are attributable to sensor dropout during QAR recording, are filled via linear interpolation within each flight. Third, each of the nine feature dimensions is standardized to zero mean and unit variance using the training-set statistics. The nine flight parameters are as follows: indicated airspeed (IAS, knots), pitch angle (degrees), roll angle (degrees), engine fan speed N1 (%), localizer deviation (dots), glideslope deviation (dots), vertical speed (ft/min), longitudinal acceleration (knots/s), and heading rate (degrees/s). No outlier removal is performed beyond the altitude gating, as the GMM-HMM with variance-floor regularization is inherently robust to moderate outliers.

To evaluate flight-level discrimination, we generate 510 synthetic deviant flights via physics-constrained perturbation of real normal flights. Three categories are synthesized, with perturbation parameters drawn uniformly from the specified ranges and applied independently per flight. Energy Imbalance, comprising 170 flights, applies vertical speed perturbations drawn from the uniform distribution U(−800,−400)∪U(400,800) ft/min, where U(a,b) denotes the uniform distribution on [a,b], with correlated N1 adjustments from U(−8%,−3%)∪U(3%,8%), where the sign of the N1 perturbation matches that of the vertical speed perturbation to preserve physical consistency. Trajectory Deviation, also 170 flights, amplifies LOC and GLIDE deviations by a multiplicative factor drawn from U(1.5,3.0) applied independently to each dimension. Attitude Anomaly, the remaining 170 flights, injects sinusoidal roll oscillations A·sin(2πft) with amplitude $A∼U(5^{\circ },12^{\circ })$ and frequency f∼U(0.05,0.15) Hz during the final 60 s of approach. All perturbations respect aircraft kinematic constraints and are injected as additive signals smoothed by a 3 s moving window with a Hamming kernel to avoid unrealistic discontinuities. The three synthetic anomaly patterns were reviewed by a senior type-rated instructor pilot, who confirmed that they faithfully reproduce the precursor signatures of real unstable approaches, including the sinker profile, lateral overshoot during intercept, and low-altitude oscillatory instability. These synthetic flights serve as a proxy for real anomalies in the absence of ground-truth anomaly labels. Because no independently labeled real-world anomaly events are available for this route, the quantitative evaluation reported in this section constitutes a controlled synthetic stress test that probes how the framework responds to physically plausible deviation patterns, rather than operational validation on real anomalous flights; this scope is revisited explicitly in the limitations. For comparative evaluation, we benchmark against three widely used unsupervised anomaly detection methods: Isolation Forest [43], Principal Component Analysis (PCA) reconstruction error, and LSTM Autoencoder (LSTM-AE). All baselines operate on the same 9-dimensional feature space.

The GMM-HMM achieves 91.2% weighted F1-score and 90.0% accuracy for 27-state SOP state inference on the 665-flight test set, consistent with the results reported in our prior work. To assess stability across data partitions, 5-fold cross-validation was conducted using stratified splits based on state-13 proportion. The model achieves a cross-validated weighted F1 of 0.885 ± 0.015 and accuracy of 0.855 ± 0.025, where the ±values denote the standard deviation across the five folds. The low standard deviations indicate that the model’s performance is stable and not an artifact of a favorable single train–test partition.

Because 93.2% of the test timesteps belong to state 13 while 18 of the 27 states are never observed in the test partition, the observation-level inference task is strongly imbalanced and aggregate measures merit additional scrutiny. On the 89,881 held-out test timesteps, macro-F1 is 0.218 and balanced accuracy is 0.405. Per-state precision, recall, and F1 are reported in Table A3, and a compact confusion matrix restricted to the operationally relevant states is given in Table A4. Recall is high for the state-13 majority at 0.935, and for the most frequent non-nominal states, sink-slow state 22 at 0.725 and the accelerating and decelerating variants of stable descent, states 14 and 12, at 0.594 and 0.565. It is lower for sink-fast state 4 at 0.161, whose samples are predominantly decoded as state 13 or state 22. This confusion concentrates along the vertical-speed axis and reflects the dominant emission ambiguity of the vertical-speed dimension reported in the component analysis. Two considerations frame these numbers. First, exact per-timestep recovery of 27 states is not the operating mechanism of the framework: deviation detection operates on continuous scores derived from the decoded sequence and from the learned reward, not on hard state assignments, and the discrimination results reported below quantify that detection task rather than the 27-way classification sub-task. Second, the per-state labels are generated by the same threshold encoding that defines the states, so per-state agreement measures the decoder’s consistency with that encoding rather than agreement with an independent operational ground truth. The metrics are nonetheless reported in full so that the difficulty of the underlying inference sub-task is transparent.

#### 3.1.1. Per-State and Per-Dimension Analysis

Figure 5 presents the decomposition of SOP compliance across four dimensions.

Panel (a) displays the sample distribution across the 27 SOP states. Of the 27 defined SOP states, 20 have at least one observation in the combined training and test dataset. However, in a stabilized approach, timesteps naturally concentrate in the nominal configuration: the aircraft should descend at near-constant vertical speed with wings level and airspeed stable for the majority of the approach. State 13, representing stable descent with heading hold and speed hold, accounts for 90.2% of all timesteps in the combined dataset and 93.2% in the test set alone, and achieves F1 of 0.953. The next most frequent states are state 4, sink-fast descent with heading and speed hold, at 3.8%; state 22, sink-slow descent with heading and speed hold, at 3.0%; state 14, stable descent with heading hold and slight acceleration, at 1.3%; and state 10, stable descent with slight left heading-rate adjustment and speed hold, at 0.6%. States 1, 3, 5, 7, 12, 16, 21, and 25 account for the remaining 0.9% of timesteps collectively. Seven states—0, 6, 8, 18, 20, 24, and 26—are never observed in either training or test data; these represent flight regimes that do not occur in the present dataset under normal approach operations. For example, state 0 combines sink-fast descent, left turn, and deceleration simultaneously—a configuration that is inconsistent with stabilized approach procedures on this route. These unobserved states serve as physical topology fences: they define the boundary of admissible configurations and prevent the Viterbi decoder from producing state trajectories that violate rigid-body kinematics.

Panel (b) shows deviation from mean values across dimensions, and panel (c) decomposes transitions by dimension. The per-dimension accuracy shows a clear operational hierarchy: lateral compliance at 99.96% and speed compliance at 99.79% are effectively at ceiling, while vertical compliance at 90.2% is substantially lower, indicating that vertical speed adjustments are the most frequent and subtle control inputs during the approach phase. State sequences decoded by the Viterbi algorithm on the test set show that 98.2% of all transitions are self-transitions, with vertical changes at 1.64%, speed changes at 0.17%, and lateral changes at 0.01%, consistent with the single-axis, single-category operational pattern encoded in the SOP transition matrix. The full distribution of empirically active states is provided in Table 1.

Panel (d) displays the quality gradient tiers. This state distribution is not an artifact of threshold selection but a direct consequence of operational practice. An approach flown according to standard operating procedure should spend the vast majority of its duration in a near-nominal configuration. The 9.8% of timesteps outside state 13 capture the procedurally informative variation: vertical speed adjustments, minor heading corrections, and airspeed modulations that constitute normal piloting behavior within the SOP envelope. The 27-state discretization resolves these subtle variations into a structured quality gradient, from the nominal state 13, through single-axis deviation states 4, 22, and 14, to multi-axis extreme states 1, 24, and 26. This gradient is what the reward function learned by IRL quantifies.

The HMM provides a principled probabilistic model of SOP state occupancy and transitions. However, state inference alone answers what the pilot did at each second; it cannot assess whether those actions constituted good SOP practice. To evaluate procedural quality, we need a reference policy that defines what the SOP-compliant pilot should do in each state. A heuristically designed reward with +10 for stable axes and −200 for extreme deviations achieves an area under the receiver operating characteristic (AUROC) of 0.685 for discriminating SOP-compliant from deviant flights, shown in Figure 6, illustrating the difficulty of specifying a reward function manually. This motivates learning the reward function directly from expert flight demonstrations via Maximum Entropy IRL.

#### 3.1.2. IRL-Learned Reward Function

The learned reward function, displayed in Figure 7, exhibits a structured three-tier quality gradient across the 27-state space. MaxEnt IRL was optimized via gradient ascent with learning rate η=0.08 for 200 iterations. The negative log-likelihood loss, the negative of the objective L in Equation (12), decreased from 1.335 at initialization to 0.056 at convergence, a reduction of 95.8%, indicating that the learned policy closely matches expert feature expectations. Convergence was smooth and monotonic, with 90% of the total loss reduction achieved within the first 80 iterations. The final feature expectation matching error, measured as the $\ell_{2}$ norm of the gradient defined in Equation (13), was 0.056, indicating that the optimization reached an effective stationary point.

Figure 8 displays the learned feature weights, with green bars indicating positive weights for SOP-compliant features and red bars indicating negative weights for deviation features. The learned weights show a structured operational hierarchy: lateral stability at $\theta_{l\text{\_}\mathrm{hold}}=+1.12$ and speed stability at $\theta_{\mathrm{sp}\text{\_}\mathrm{hold}}=+1.12$ are each weighted 1.6× above vertical stability at $\theta_{v\text{\_}\mathrm{stable}}=+0.69$. A feature ablation experiment shows that removing the is_nominal feature preserves the state reward ranking perfectly, with Kendall τ=1.00, demonstrating that the structure learned by IRL is a multi-dimensional quality assessment rather than a single-state classifier.

The learned reward R(s) defines a three-tier quality gradient across the 27-state space:

1. Nominal tier (R≈+5.52): State 13 alone, where all three axes are at their nominal categories. The $\theta_{\mathrm{is}\text{\_}\mathrm{nominal}}$ feature contributes +2.59 on top of the already-positive axis contributions, reflecting that the exact nominal configuration carries the highest operational value: it is the target state that the entire approach procedure is designed to maintain.
2. Near-nominal tier (R≈+1.25 to +1.90): States with two stable axes and one non-extreme deviation. State 4 (sink-fast, +1.91) and state 22 (sink-slow, +1.88) form the upper boundary of this tier with only a vertical deviation; state 14 (stable + accelerating, +1.25), state 12 (stable + decelerating, +1.25), state 10 (stable + slight left heading, +1.25), and state 16 (stable + slight right heading, +1.25) form the lower boundary with only a lateral or speed deviation. The reward difference between states 4/22 and 14/12/10/16 (≈+0.65) reflects the IRL’s learning that vertical deviations are more common and less penalized than lateral or speed deviations in expert behavior.
3. Extreme tier (R≈−1.48 to +0.23): States with two or more non-nominal axes, or with any axis at an extreme category. These states are rarely visited in expert trajectories and receive negative or near-zero rewards, serving as a learned penalty boundary that the optimal policy avoids.

The IRL recovers a graded quality ordering in which the operational severity of a deviation, including which axis and how far from nominal, is reflected in the reward value. Figure 7b–d displays the state value function $V^{*}\left(s\right)$ organized by dimensional decomposition, with each subplot showing lateral category (rows) by speed category (columns) for one vertical category. The value structure mirrors the reward gradient: states in the nominal tier have $V^{*}\approx 112.33$; near-nominal states with two stable axes cluster tightly at $V^{*}\approx 112.25$; and extreme states converge to $V^{*}\approx 104.58$–108.97, depending on the number of stable axes. From 24 of 27 states, the optimal policy directs transitions toward state 13, which serves as the SOP-compliant attractor.

The cross-definition replication with smoothness-based expert selection showed similar convergence behavior: loss decreased from 1.689 to 0.044, a reduction of 97.4%, with a final gradient norm of 0.044. The learned reward achieves Kendall τ=0.91 with the original reward despite only 6.2% overlap in which flights are labeled expert, indicating that the quality gradient is consistent across expert definitions.

### 3.2. Compliance Scoring

The IRL-Hybrid method achieves an AUROC of 0.941 on the synthetic deviant test set, outperforming the manually defined MDP baseline by +0.243. Across all test flights, 9.9% of transitions exhibit a compliance gap exceeding 0.1. Among dimension-changing transitions with a compliance gap, vertical deviations account for 86.4%, followed by speed at 9.7%, lateral at 0.7%, and multi-axis at 3.2%. Single-axis corrections thus account for 96.8% of dimension-changing gaps, with vertical speed management as the dominant procedural concern. Figure 9 compares expert and non-expert score distributions for the value score (panel a) and compliance gap (panel b). Transition-level compliance is more discriminative than state-level value alone (Cohen’s d=1.63 vs. 0.73 for the value score).

To contextualize the IRL contribution, we compare against three statistical baselines computed directly from the HMM state sequences without any reward learning: (i) state-13 occupancy fraction, the simplest possible compliance proxy; (ii) transition rate, the normalized count of state changes per flight; and (iii) state entropy, measuring the diversity of visited states. Table 2 reports their flight-level discrimination performance against the same synthetic deviant test set.

The state-13 fraction achieves AUROC 0.926, indicating that the 27-state encoding captures procedurally relevant structure without learning. State entropy achieves AUROC 0.936, outperforming state-13 fraction and supporting the multi-state perspective that the diversity of visited states carries discriminative information. The IRL-Hybrid reaches AUROC 0.941. The gain over the best learning-free baseline is therefore small, at +0.005 in AUROC, and discrimination performance is not the primary rationale for introducing the IRL component. Beyond discrimination performance, the IRL framework provides three capabilities that simple metrics cannot: a state-level reward function *R*(*s*), a per-timestep compliance gap metric, and a counterfactual engine for procedural diagnosis.

For flight-level discrimination, the transition NLL score attains the highest F1 among the methods compared in Table 3, reaching 0.893 against 0.869 for IRL-Hybrid, indicating that SOP state transition consistency is the strongest pure discriminative signal. The IRL framework serves a qualitatively different role: it provides a reward function *R*(*s*) that quantifies each state’s operational value, a per-timestep compliance gap metric, and a counterfactual engine, capabilities beyond what a generative-model likelihood provides. For a continuous leading indicator, discrimination alone is insufficient; the ability to explain why a flight deviates and what corrective action to take is equally essential. More generally, classification accuracy and interpretability are distinct objectives: a higher F1 likelihood score identifies which flights are anomalous but yields no decomposition of the deviation, whereas the IRL score trades a small amount of discrimination for a reward function, a compliance gap, and corrective guidance that a safety analyst can act on. In an operational setting, the two are complementary and are intended to be used together. The full scoring method comparison is provided in Table 4 and Figure 10.

### 3.3. Component Analysis and Structural Ablation

The three auxiliary components serve distinct purposes and are evaluated on their own objective functions: emission regularization informed by physics (A), split conformal calibration (B), and counterfactual explanation (C). Regularization informed by physics improves synthetic deviant AUROC by +0.0047 systematically across all configurations containing A, a significant effect at p<0.01 by permutation test, with negligible impact on state F1 of −0.0003. Split conformal calibration achieves empirical false alarm rate (FAR) of 4.2–4.4% across all configurations containing B, within the 5% theoretical bound. Calibration set size sensitivity analysis shows that $n_{\mathrm{cal}}\geq 100$ is sufficient for stable FAR estimation. Counterfactual explanation identifies 6.4% of timesteps as procedural deviations, with all top-5 corrections involving the vertical axis; detailed results are presented in Section 3.4.

#### 3.3.1. Structural Ablation of the SOP Transition Constraint

To quantify the contribution of the transition structure informed by the SOP, we replace the learned transition matrix with three alternative variants while keeping the GMM emission models, which are trained on SOP-labeled data, unchanged: (i) uniform, where all 729 transitions are equally probable at 1/27, representing complete ignorance of procedural structure; (ii) unconstrained, estimated from data, where transition probabilities are estimated from training labels without enforcing the single-axis, single-category SOP filter, with 586 of 729 transitions observed; and (iii) biased toward inertia, a generic smoothness prior with 90% self-transition probability.

Table 5 reports the structural impact of removing the SOP constraint. The uniform transition matrix reduces weighted F1 from 0.912 to 0.852 (6.6% drop), but the GMM emissions already carry substantial discriminative information. The data-estimated unconstrained variant achieves essentially identical F1 (0.9123 vs. 0.9121) but admits 119 SOP-invalid transitions (16.3% of transition space), undermining procedural interpretability. The SOP-constrained model uniquely provides both high accuracy and zero procedurally invalid transitions, guaranteeing that every inferred state sequence respects the single-axis, single-category operational principle. The SOP constraint’s primary value is therefore structural rather than discriminative: it guarantees operationally meaningful transitions, a prerequisite for downstream compliance quantification.

#### 3.3.2. IRL Feature Decomposition

The linear reward parameterization $R\left(s\right)=\theta^{⊤}\phi \left(s\right)$ makes it possible to quantify the contribution of each feature to the learned state ordering. The is_nominal feature, active only for state 13, contributes $\theta_{\mathrm{is}\text{\_}\mathrm{nominal}}=+2.59$ to the reward. To verify that the nine axis features carry information beyond this single binary indicator, we re-trained MaxEnt IRL with the is_nominal feature removed, leaving only the nine per-axis stability and deviation features.

Removing the is_nominal feature while retaining the nine axis features produces Kendall τ=1.00 against the full model with identical top-5 states. The three axis-stability features alone achieve τ=0.90, preserving the top-3 states and quality structure. Vertical features alone drop to τ=0.34, indicating that multi-dimensional features are essential. The dominance of vertical-axis counterfactual corrections is explained by GMM emission variance: V_SPD_ standard deviation is approximately 63.2 in standardized units, roughly 20–120× larger than lateral features (LOC: 3.2, GLIDE: 0.5) and speed features (IAS: 2.0, ACC: 1.0). Vertical speed is the most observationally ambiguous dimension in the present data, and the counterfactual engine correctly reflects this data-level uncertainty.

### 3.4. Counterfactual Explanation and Case Illustration

Across all test flights, the counterfactual engine identifies a mean of 8.6 deviations per flight, or 6.4% of approach timesteps, with a mean per-step deviation cost of 0.0094. The low cost indicates that even when the actual state diverges from the counterfactual, the SOP-optimal state is nearly as plausible as the observed state given the flight data. All top-5 counterfactual corrections involve the vertical dimension: S04→S13 (47.0%), S22→S04 (30.9%), S22→S13 (16.4%), S04→S22 (1.4%), and S14→S13 (1.4%). The full top-5 counterfactual corrections are listed in Table 6. This vertical concentration reflects the finding that vertical speed ambiguity is the dominant source of SOP deviation, consistent with the per-dimension decomposition discussed above.

To illustrate the framework’s counterfactual explanation capability, Figure 11 presents the actual versus optimal state trajectory for a representative flight drawn from the held-out test set. The optimal trajectory is computed by solving the Markov decision process (MDP) under the IRL-learned reward function via value iteration, producing the state sequence that maximizes cumulative reward. This comparison identifies specific moments where the pilot’s actions diverged from the optimal SOP-compliant sequence, providing actionable procedural feedback that is invisible to threshold-based monitoring.

Over the 131-second approach (Figure 11a), the optimal MDP policy assigns the nominal state 13 at all 131 timesteps, reflecting the reward function’s strong preference for the nominal SOP configuration (state 13 receives the highest reward of +5.52). The pilot’s HMM-inferred state sequence matches this optimal path at 110 of 131 timesteps (84.0%), with a policy gap of 0.122, and diverges at 21 timesteps. These deviations briefly enter sink-fast states S3 and S4, and stable variants S12 and S14, concentrated in two clusters: an early sink-fast excursion through state 4 around t≈10–20, and a later phase with alternating visits to states 12, 14, and 22 around t≈90–110.

The reward gap (Figure 11b) quantifies the procedural cost of each deviation as $R\left(s_{t}^{*}\right)-R\left(s_{t}\right)$, where $s_{t}^{*}$ is the optimal state and $s_{t}$ the actual state. The largest gap (≈5.29) occurs at a deviation to state 3, while sink-fast excursions to state 4 incur gaps of R(13)−R(4)≈3.61; both reflect the reward penalty assigned to vertical-speed departures from the stable descent profile. The mean reward gap of 0.62 across all timesteps provides a per-second measure of procedural departure from optimality.

This counterfactual analysis constitutes the third component of the framework’s safety performance indicator (SPI) suite: given an observed state sequence, the IRL-learned reward function identifies which timesteps deviated from optimal SOP execution, by how much, and what the preferred alternative would have been. Traditional FDM threshold-based monitoring would report zero exceedances for this flight, providing no visibility into the procedural structure that the framework captures.

### 3.5. Comparative Evaluation, Stability, and Sensitivity Analysis

To evaluate robustness, Gaussian noise $N(0,\sigma^{2}\cdot \mathrm{var}\left(f\right))$ is injected into each feature dimension at intensities σ∈{0.00,0.05,0.10,0.15,0.20,0.25}. As shown in Figure 12, the framework maintains stable observation-level F1-score across the full noise range, with degradation below 0.012. This robustness is consistent with two regularizing mechanisms in the model design: the variance floor ($10^{-4}$) applied to GMM covariances, which prevents emission models from overfitting to precise feature values, and the SOP transition constraint, which restricts the state space to operationally plausible sequences.

The framework demonstrates data efficiency: at 10% of training data (266 flights), state inference F1 reaches 0.885, representing 97% of the full-data performance compared to 0.912 at 2660 flights, as shown in Figure 12b. This efficiency is enabled by the SOP structure: the transition matrix encodes strong domain knowledge that reduces the effective degrees of freedom the model must learn from data. The GMM component count was set to K=2, chosen as the balanced trade-off between aggregate inference accuracy and minority-state detection, shown in Figure A2.

#### 3.5.1. Comparison with Baseline Methods

The framework achieves the best flight-level F1 of 0.893 and observation-level F1 of 0.559 while providing per-state interpretability, a capability not provided by the baseline methods considered, as shown in Table 7 and Figure 13 (panels a and b report observation-level and flight-level F1, respectively). LSTM-AE achieves competitive flight-level F1 of 0.886 but outputs only a scalar reconstruction error without explaining which procedural element was anomalous. The observation-level F1 ceiling of 0.56 reflects the inherent difficulty of the per-timestep task: with 93.2% of samples in state 13, a model predicting normal everywhere achieves 93.2% accuracy but zero F1 on minority classes. Cross-comparison of robustness and data efficiency across methods is shown in Figure A1.

To further verify that the framework captures physically distinct deviation patterns, we evaluate per-type detection performance. Trajectory Deviations are detected most reliably (AUROC = 0.954 via gmm_nll), followed by Energy Imbalance (AUROC = 0.905) and Attitude Anomaly (AUROC = 0.877). The lower detection rate for attitude anomalies is attributable to their limited sample size after altitude filtering and the inherent variability of roll behavior near the ground. The full per-type metrics are reported in Table A1.

#### 3.5.2. Stability Across Expert Definitions and Model Configurations

To verify that the learned reward structure is independent of expert selection criteria, we re-selected expert flights using an entirely orthogonal criterion: physical QAR smoothness, defined as the negative weighted variance of raw flight parameters (IAS, pitch, roll, N1, LOC, GLIDE, V_SPD_, ACC, heading rate). This criterion makes no reference to the 27-state SOP encoding.

The physical smoothness criterion and state-13 fraction are moderately correlated, with Pearson r=0.52 and Spearman ρ=0.38 at $p<10^{-90}$, indicating they capture related but distinct aspects of flight quality. Only 6.2% of flights are selected as experts by both criteria. The IRL re-run with smoothness-based experts yields a reward function strongly correlated with the original: Kendall τ=0.91 at $p=9.5\times 10^{-18}$, with Spearman ρ=0.98 and Pearson r=0.99. The two expert definitions thus yield consistent state value rankings. The 27-state discretization captures a quality gradient independently recoverable through a physically grounded signal, indicating that the author-chosen thresholds reflect operational structure rather than an artifact of the encoding. The procedural optimum and the physical optimum coincide because good SOP practice produces physically smooth flight. Bootstrapping with 1000 resamples yields a 95% confidence interval of [0.43,0.58] for Cohen’s *d* between smoothness-based experts and non-experts, indicating statistically significant separation.

The apparent paradox of 6.2% expert overlap yet Kendall rank correlation 0.91 is resolved by the MaxEnt IRL objective structure: the algorithm matches expected feature counts aggregated across the entire expert set, as formalized in Equation (12), not individual trajectories. Two expert sets with minimal overlap produce nearly identical aggregate feature statistics because both criteria, consistent SOP execution and smooth physical control, correspond to the same direction of improvement in state-occupancy space, toward state 13. This property is practically significant: an airline can define experts by its own operational criteria and expect the IRL to recover a consistent reward structure.

The learned reward is also stable under variation in the expert selection threshold and HMM configuration. Varying the expert percentile from 10% to 50% yields pairwise Kendall rank correlation above 0.91 with stable clusters at 10–15% and 20–50%. Varying the variance floor (0.1×–10×) and GMM components (K∈{1,2,3,4}) shifts weighted F1 within only 0.023 (0.888–0.911), indicating that the HMM is a stable component.

A hyperparameter sensitivity analysis (S1–S8) shows that the reward function learned by IRL remains stable across the tested hyperparameter ranges, with learning rate being the only parameter requiring non-trivial selection, as summarized in Table A2. Reward ranking stability is quantified by the Kendall rank correlation coefficient *τ* between the reward vectors produced by different configurations.

We verified that the default T=200 iterations provide adequate convergence by running MaxEnt IRL to T=500 with checkpoints every 10 iterations, computing Kendall *τ* between the reward at each checkpoint and the fully converged reference at T=500. The reward ranking stabilizes rapidly: *τ* reaches 0.818 by iteration 40 and remains stable through iteration 500, with 90% of the total loss reduction achieved within the first 80 iterations. At T=200, the reward achieves τ=0.818 with the fully converged reference, and the qualitative findings (state 13 ranked first, the top-5 state set, and the expert/non-expert discrimination) are fully preserved.

To assess sensitivity to random initialization, we tested 15 independent random seeds {0,1,2,3,4,5,6,7,8,9,42,123,456,789,1024} for the reward parameter vector $\theta_{0}∼0.01\cdot N\left(0,I\right)$. Across 15 seeds, the mean pairwise Kendall *τ* between reward vectors was 0.892 with standard deviation 0.052. State 13 was the highest-ranked state in all 15 seeds. The consensus top-5 states were {13,4,22,3,5}, with Cohen’s *d* ranging from 0.483 to 0.500 (mean 0.492 ± 0.007). The key structural properties—state 13 as the optimal state, with states 4 and 22 in the top 3—are perfectly preserved across all 15 seeds.

#### 3.5.3. Optimization Hyperparameters

The discount factor *γ* was swept across {0.70,0.75,0.80,0.85,0.90,0.92,0.95,0.97} while holding all other hyperparameters fixed. The top-5 states {13,4,22,12,16} were identical across all eight values, and $\theta_{\mathrm{nominal}}$ varied only 0.4% (within [1.5748,1.5810]). Cohen’s *d* decreased monotonically from 0.528 at γ=0.70 to 0.498 at γ=0.97, a negligible change of 0.03 units.

The learning rate *η* is the only hyperparameter requiring non-trivial selection. Across η∈{0.005,0.01,0.02,0.05,0.08,0.10,0.15,0.20,0.50}, three regimes emerge: (i) η≤0.01 fails to reach the convergence basin within the allocated iterations; (ii) η∈[0.02,0.10] produces identical top-5 states and stable Cohen’s *d* of 0.50–0.52; (iii) η≥0.15 inflates $\theta_{\mathrm{nominal}}$ to 1.74–2.04 and shifts the top-5 ranking. The baseline η=0.08 lies at the center of the stable region.

The soft value iteration (inner loop) parameters—convergence tolerance $\varepsilon_{\mathrm{VI}}\in \left\{10^{-4},\ldots ,10^{-12}\right\}$ and maximum iterations $K_{\max}\in \left\{500,\ldots ,5000\right\}$—and the visitation horizon H∈{50,…,1000} are all inconsequential: every configuration tested produced τ=1.0 with the baseline reward and identical top-5 states. A 3×5 grid sweep of γ×η confirms that learning rate is the sole bottleneck: η=0.01 always fails (τ<0.85) regardless of *γ*, while η≥0.02 always succeeds (τ=1.0) regardless of *γ*, with no interaction effect requiring joint tuning.

We additionally verified two theoretical properties of the MaxEnt IRL framework. First, reward normalization (rescaling to zero mean and unit variance) preserves the rank ordering perfectly (τ=1.0, Spearman ρ=0.999). Second, potential-based reward shaping with a state-dependent potential function Φ(s) defined from geographic distance or altitude deviation does not preserve the optimal policy (τ<0.85 compared to the unshaped reward).

In summary, the reward function learned by IRL is stable across all hyperparameters within reasonable ranges, with learning rate being the only parameter requiring non-trivial selection. The discount factor, VI tolerance, visitation horizon, and reward normalization have negligible impact on the learned reward ordering.

#### 3.5.4. Amplitude Sensitivity and Detection Latency

To characterize how detection behaves as a deviation becomes more or less pronounced and how promptly the per-timestep signals respond, we generated fixed-severity synthetic deviations across an amplitude grid s∈{0.4,0.6,0.8,1.0,1.2,1.4}. The amplitude multiplier *s* scales the perturbation of each deviation class relative to the magnitudes used for the 510-flight test set, and a deviation is retained only if it reduces the state-13 occupancy of the perturbed segment by more than 5%, the same retention criterion as the original test set. Detection degrades gracefully as the amplitude falls, as shown in Figure 14. For the energy-imbalance class, whose drift is gradual, the flight-level AUROC of the IRL-hybrid score declines from 0.980 at s=1.4 to 0.800 at s=0.4 and remains well above chance at every tested amplitude. The attitude-anomaly class, whose heading-rate excursion is present from the first perturbed timestep, is detected at AUROC 0.973 to 0.982 across the whole grid. The trajectory-deviation class produces a measurable change in SOP-state occupancy only for s≥1.0, where its AUROC reaches 0.974 to 0.994. Pooled over classes, the IRL-hybrid AUROC at the reference amplitude s=1.0 is 0.958 for n=392, consistent with the value of 0.941 reported on the 510-flight test set.

For reference, we also applied a conventional threshold-based exceedance monitor that alarms whenever any monitored parameter crosses a fixed operating limit. Because every synthetic deviation is constrained to remain inside the operating envelope, that monitor issues no alarm on the retained deviations at any amplitude, giving 0 out of *N*, so its output carries no discriminative information, as shown by the gray dashed line in Figure 14.

To quantify how promptly the framework flags a developing deviation, we measured the number of timesteps from the deviation onset to the first timestep at which the inferred SOP state departs the nominal configuration. This first procedural flag is provided by the calibration-free IRL policy-gap signal and fires on every retained deviation. At the reference amplitude s=1.0, the median time to flag is 3 timesteps, with an interquartile range of 0 to 15, for energy-imbalance deviations, 2 timesteps for trajectory deviations, and 0 timesteps for attitude anomalies, whose perturbation departs the nominal state at the onset step itself. Table 8 reports the latency results for all three classes. For the gradually drifting energy-imbalance class, the flag time shortens as the deviation becomes more pronounced, from a median of 11 timesteps at s=0.8 to 1 timestep at s=1.2. The continuous per-step scores respond over the same window: at a per-step threshold exceeded by only 0.1% of normal timesteps, the IRL value and transition-NLL signals flag 80% and 66% of the retained deviations at s=1.0, respectively. Because the perturbation protocol operates strictly inside the operating envelope, a conventional exceedance alarm never fires on these deviations, so a lead time relative to such an alarm is not defined in this regime. The framework instead provides a graded score that begins to depart from the normal range as soon as a deviation becomes procedurally identifiable, and it flags the deviation at the first departure of the SOP-state trajectory from nominal.

## 4. Discussion

### 4.1. From Exceedance Detection to Deviation Quantification

The 27-state discretization resolves approach execution into a structured quality gradient, from nominal (state 13) through single-axis deviations to multi-axis extremes. That state 13 accounts for 90.2% of timesteps is a domain property of stabilized approaches; the procedurally informative variation resides in the remaining 9.8%, where pilots execute necessary corrections within the procedural envelope. The per-dimension decomposition shows that procedural variation concentrates in the vertical axis: vertical compliance is 90.2%, while lateral and speed compliance both exceed 99.7%. This provides concrete targets for training interventions and monitoring priorities. The operational relevance of vertical-axis vulnerability is consistent with documented turbulence risk on comparable Asian routes, where deep convective clouds can produce vertical accelerations sufficient to cause injuries and structural risk [44].

Beyond the structural prior encoded in the SOP transition matrix, the framework incorporates two complementary mechanisms that strengthen its reliability without requiring anomaly labels. The emission regularization informed by physics, applied with strength 0.05, improves AUROC by 0.0047 systematically across all configurations without disrupting the EM learning objective. The split conformal prediction module addresses a fundamental challenge in flight safety analytics, the absence of real anomaly labels, by providing a false alarm rate guarantee that requires only a calibration set of normal flights. At the 5% significance level, the empirical false alarm rate of 4.2% is within the theoretical 5% bound. The conformal guarantee holds for flights exchangeable with the calibration set; if the operational environment changes substantially, re-calibration requires only normal-flight data and no anomaly labels.

### 4.2. Learned Reward: Empirical Validation and Structural Stability

The IRL-based scoring achieves AUROC 0.941, exceeding the manually defined MDP reward at AUROC 0.685. The learned reward function correctly weights lateral and speed stability 1.6 times above vertical stability, whereas the manually defined function assigned penalty values that appeared reasonable but did not reflect the operational structure present in the data. Weights recovered from expert demonstrations capture operational structure not captured by heuristic rules.

The IRL reward function remains stable across multiple verification axes. Cross-definition expert selection yields Kendall rank correlation 0.91 despite 6.2% expert overlap; varying the expert percentile from 10% to 50% produces pairwise correlation above 0.91; and HMM hyperparameter variation shifts weighted F1 within only 0.023. This multi-level stability is not assumed and indicates that the 27-state encoding and MaxEnt IRL objective jointly yield a stable learning problem whose solution is insensitive to reasonable variation in modeling choices.

The convergence of the cross-definition replication, expert threshold insensitivity, and feature ablation on the same conclusion reflects three properties of the MaxEnt formulation. The maximum entropy objective commits to the minimally committal distribution consistent with observed feature expectations, so the learned reward is the simplest function consistent with the expert data. The linear parameterization with 10 interpretable features limits model capacity and prevents overfitting. The convexity of the MaxEnt objective guarantees that any stationary point of sufficient accuracy is close to the global optimum. These properties indicate that the learned reward ordering is consistent across the tested expert definitions and modeling choices.

The reward function includes an explicit indicator for state 13, and experts are selected by the fraction of time spent in state 13, so the valuation assigned to the nominal configuration could in principle follow from that definition alone. Three independent analyses distinguish the two. First, the reward ranking is recovered without the nominal indicator: removing that feature and re-training on the nine per-axis features alone preserves the state ordering perfectly at Kendall τ=1.00, so the valuation of the nominal configuration is carried jointly by the axis features and is not contingent on a dedicated nominal-state indicator. Second, an entirely orthogonal expert criterion, physical QAR smoothness, which makes no reference to the SOP encoding, selects a flight set that overlaps the state-13 criterion by only 6.2% yet yields a reward ranking correlated with the original at Kendall τ=0.91. Third, the learned ordering is insensitive to the expert threshold across the 10th to 50th percentile range. Together these analyses indicate that the learned reward reflects a quality gradient recoverable from both procedurally grounded and physically grounded signals. We therefore interpret the IRL component as learning an operational value function that is consistent with, but not reducible to, the state-13-based definition of compliance.

### 4.3. SMS Integration, Limitations, and Future Work

The proposed framework was designed to function as a component of an operational Safety Management System, and its architecture reflects this intent. Each layer maps onto established aviation safety requirements. The linear reward parameterization provides the explainability that Ahangar et al. [45] identify as essential for trustworthy AI in quality-critical monitoring systems; split conformal prediction supplies complementary distribution-free statistical guarantees; regularization informed by physics enforces physical consistency; and the SOP-constrained transition matrix confers robustness to distribution shift. Within the ICAO SMS framework defined in Annex 19 [4], the GMM-HMM corresponds to safety data collection and processing, the Q-value compliance gap provides a quantitative basis for safety performance monitoring, conformal calibration supports safety risk management through bounded false alarm rates, and counterfactual explanations contribute to safety promotion. Rather than adding these as supplementary features, we built the framework so that the SPI, the statistical guarantee, and the explanation share a single MDP value function, making the safety case consistent across layers. Beyond explainability, uncertainty-aware deep learning with adjustable decision thresholds has been proposed for trustworthy civil aviation hazard identification, decomposing predictive uncertainty into epistemic and aleatoric components [46]. In operational deployment, the framework would sit alongside existing FDM exceedance detection as a complementary post-flight analysis layer, consuming the same QAR data stream and requiring no changes to existing data collection pipelines.

The empirical scope of this validation spans two Airbus aircraft families, the A319 and A330, and multiple operators on a single high-plateau route. The evaluation is therefore not confined to one aircraft model or one operator, and the shared FCOM-based category definitions mean that the framework does not encode operator- or model-specific scoring rules. Per-family and per-operator breakdowns are not reported, so generalization across aircraft families and operators rests on the breadth of the evaluation data rather than on controlled cross-context experiments, and we characterize the reported results as a proof of concept at the framework level. Extending the framework to an additional aircraft type, route, or flight phase would require re-estimating the SOP category thresholds from that context’s flight crew operating manuals and operational data and re-calibrating the conformal threshold on normal flights from the new environment, because the distribution-free guarantee in Equation (20) holds only for flights exchangeable with the calibration set. Re-calibration requires only normal-flight data and no anomaly labels, which limits the cost of deployment in a new operational context.

Several limitations of the current implementation warrant acknowledgment. First, validation of the flight-level discrimination and of the deviation diagnosis relies on synthetically generated deviant flights rather than on independently labeled real-world anomaly events, because the assembled QAR corpus for this route contains no exceedance events or expert-flagged unstable approaches. The perturbation classes were designed to reproduce the precursor signatures of documented unstable-approach phenomena and were reviewed by a senior type-rated instructor pilot. Synthetic deviations may not capture the full complexity and diversity of real operational deviations, so the reported metrics should be read as the outcome of a controlled synthetic stress test rather than as operational validation. Second, the amplitude-sensitivity and detection-latency analysis in Section 3.5.4 is confined to the sub-threshold regime, in which conventional threshold monitoring issues no alarm by construction; a quantitative comparison of detection lead time against conventional threshold monitoring on data that contain genuine exceedance events is therefore left to future work. Third, vertical speed management is identified as the dominant source of procedural deviation, but the present data cannot determine whether this reflects pilot workload, route-specific atmospheric conditions, aircraft energy state, or simply the width of the vertical-speed category boundaries; physiological or subjective workload measures would be needed to resolve this attribution. Fourth, the expert demonstration selection criterion uses state-13 occupancy as a proxy for pilot expertise, which conflates pilot skill with operational difficulty; the cross-definition and sensitivity analyses mitigate this by demonstrating stability under alternative expert definitions at Kendall τ≥0.91. Counterfactual explanations lack a ground-truth reference for procedural correctness and would benefit from pilot-in-the-loop evaluation to establish operational validity. Future work includes nonlinear reward parameterization via neural IRL to capture higher-order feature interactions, a quantitative lead-time comparison against conventional threshold-based monitoring on data containing documented exceedance events, pilot-in-the-loop evaluation of counterfactual explanations, and online deployment for real-time approach monitoring.

## 5. Conclusions

This paper presents a framework for continuous operational deviation quantification based on flight data. The framework combines SOP-constrained GMM-HMM state inference with maximum entropy IRL for expert value function estimation, split conformal prediction for distribution-free statistical guarantees, and counterfactual explanation for procedural diagnosis. Its formulation does not depend on a specific aircraft type, route, or dataset, and it requires no anomaly labels beyond SOP state definitions. The results answer the three research questions posed in the Introduction. Continuous quantification of procedural execution quality is feasible from QAR data alone, giving visibility into gradual deviation that threshold-based monitoring leaves unseen. The reward function recovered by maximum-entropy IRL provides an interpretable valuation of the procedural states that is stable across expert definitions and consistent with independent physical signals. Split conformal prediction bounds the false alarm rate of the resulting score, and counterfactual analysis localizes each detected deviation to its procedural origin. Each of these findings is developed in turn below.

The 27-state SOP encoding defines a meaningful quality gradient. State occupancy entropy alone achieves AUROC 0.936, confirming that the discretization captures substantial operational structure without any learning. The IRL-learned reward function further differentiates this structure through multiple lines of evidence. Cross-definition expert selection yields Kendall rank correlation 0.91 despite only 6.2% expert overlap; expert threshold sensitivity maintains correlation above 0.91 across thresholds from 10% to 50%; and feature ablation confirms that the three per-axis stability features encode the same quality ordering independently of any binary nominal-state indicator. The learned reward weights lateral and speed stability 1.6 times above vertical stability, reflecting expert behavioral patterns recovered from observational data.

Split conformal prediction controls the false alarm rate with distribution-free guarantees, achieving an empirical false alarm rate of 4.2% under a nominal 5% significance level. Counterfactual explanations ground procedural feedback in the learned policy: 6.4% of timesteps are identified as procedural deviations, with all top-5 corrections involving the vertical axis, confirming this dimension as the dominant source of operational variation.

Deviation scoring, statistical calibration, and procedural diagnosis are integrated within a single framework through a shared MDP value function, forming a candidate continuous safety performance indicator that provides visibility into procedural execution quality before any parameter reaches an exceedance boundary; quantifying the corresponding detection lead time against conventional threshold monitoring on data containing documented exceedance events is left to future work.

## Figures and Tables

**Figure 1:**
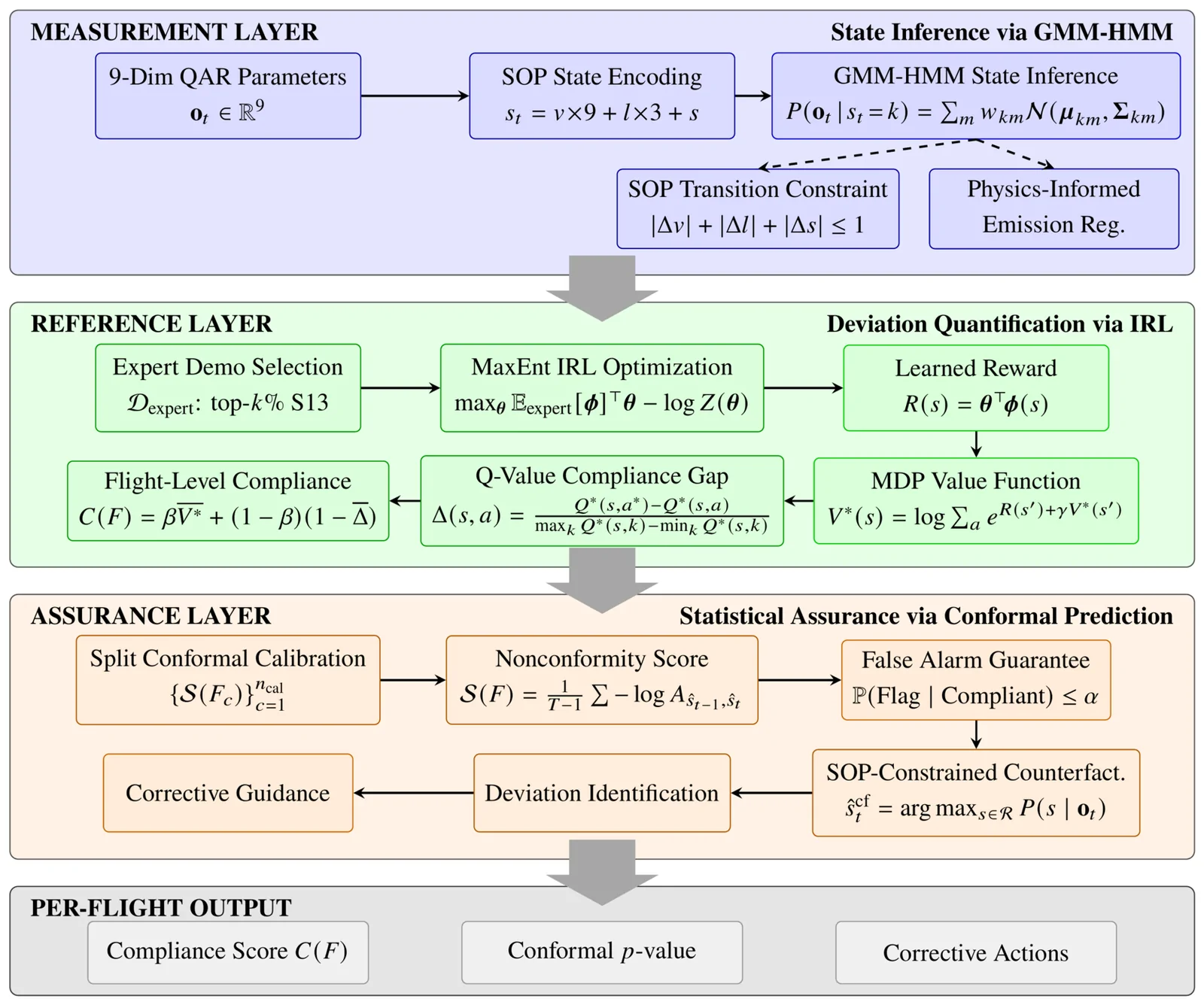
Three-layer architecture of the proposed operational deviation quantification framework. The arrows trace the flow of information across the three layers, from state inference through value learning to statistical assurance and the per-flight output.

**Figure 2:**
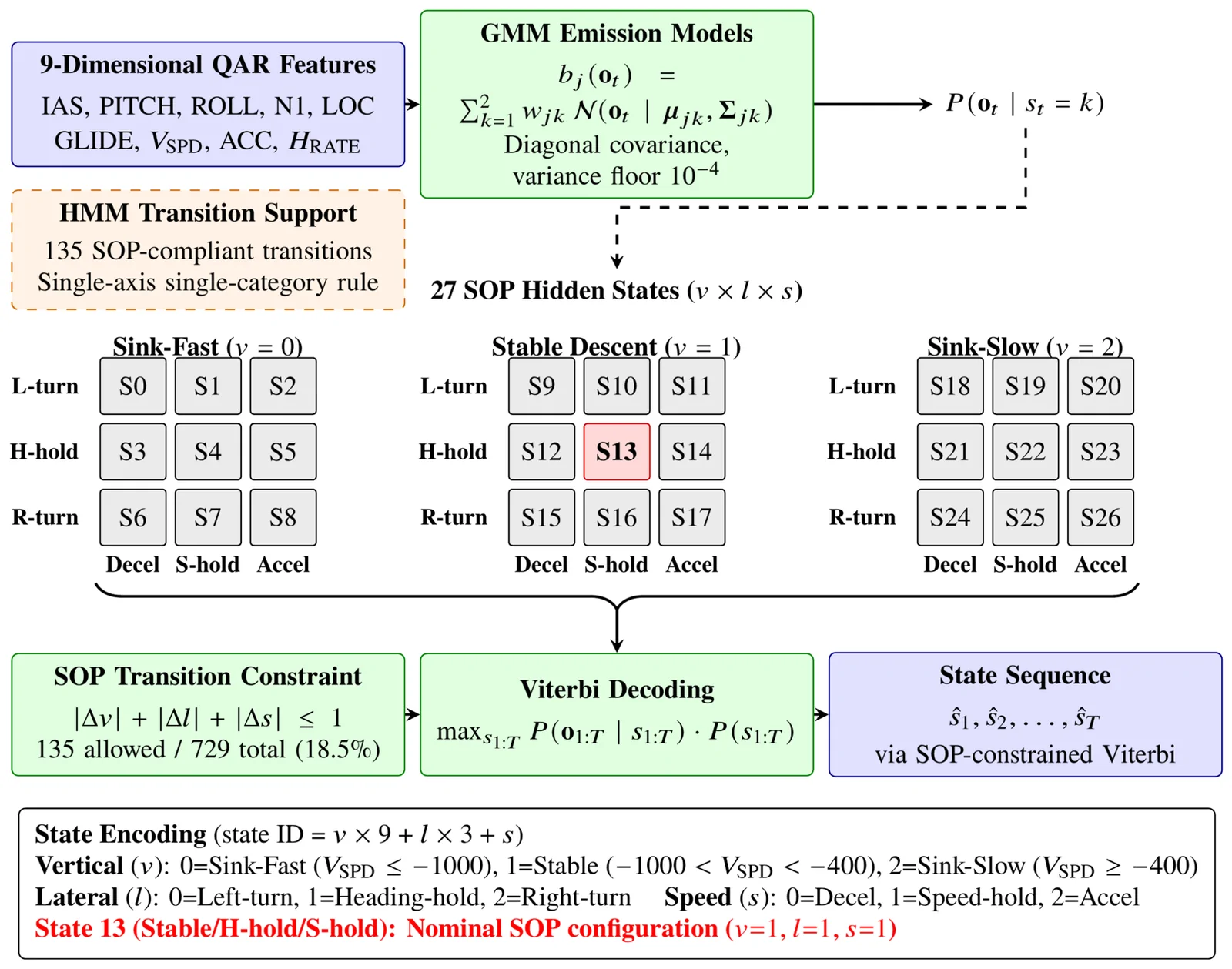
GMM-HMM architecture for SOP state inference. Blue boxes denote flight data, green boxes the model components, orange boxes the transition support, and the red cell the nominal state 13; the arrows trace the inference flow from observations to the decoded state sequence.

**Figure 3:**
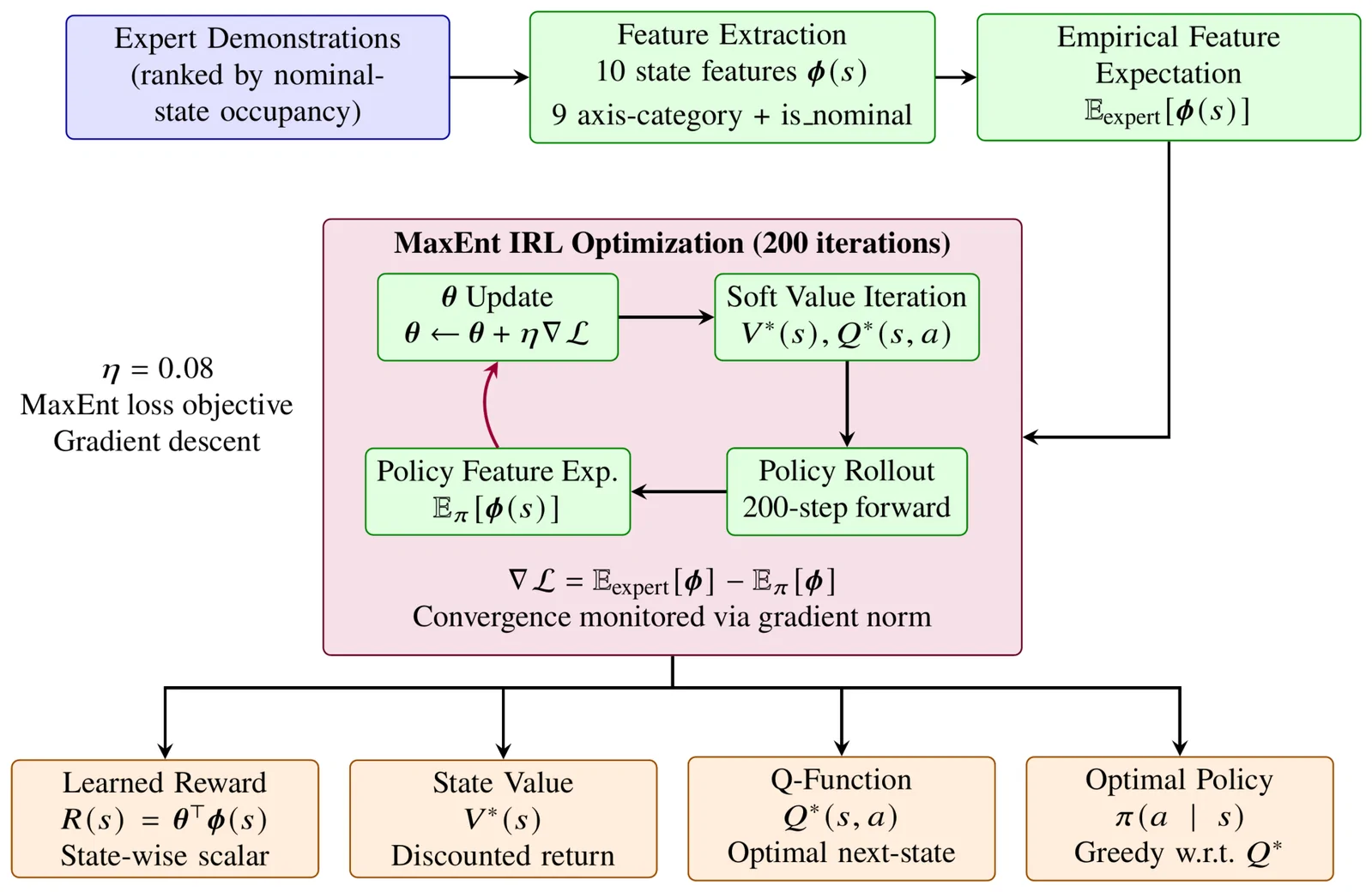
Maximum entropy IRL pipeline for reward function learning. Blue boxes denote inputs, green boxes processing steps, and orange boxes the derived quantities; the black arrows trace the forward pipeline and the purple arrow the iterative update of the reward parameters.

**Figure 4:**
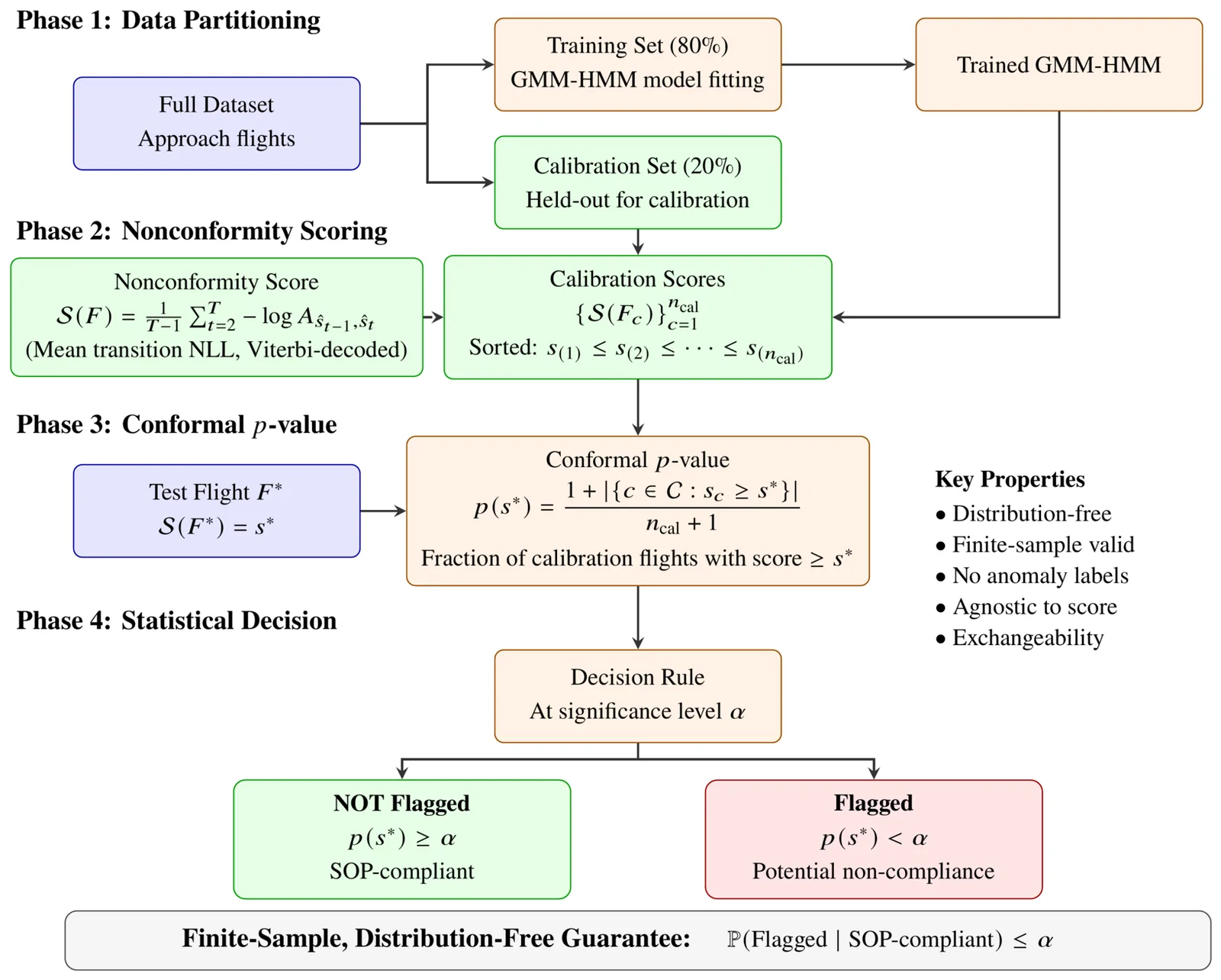
Split conformal prediction workflow for distribution-free calibration of compliance thresholds. Blue boxes denote data, orange boxes processing steps, green boxes calibration and the SOP-compliant outcome, and the red box the potential non-compliance outcome; solid arrows trace the workflow and the dashed arrow carries the nonconformity scores into the calibration set.

**Figure 5:**
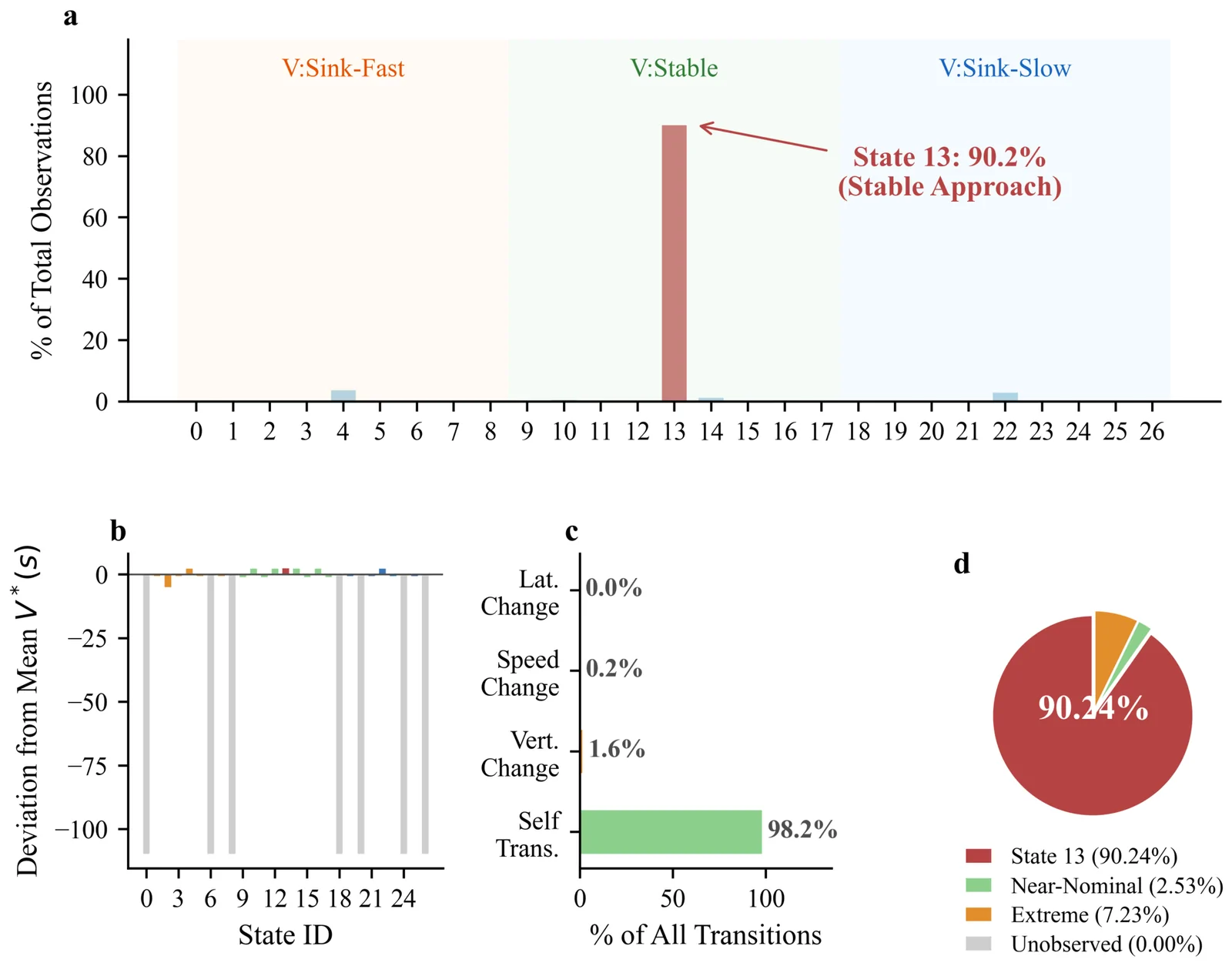
SOP compliance decomposition. (**a**) Sample distribution. (**b**) Deviation from mean value. (**c**) Transition dimension composition. (**d**) Quality gradient tiers. In panels (**a**,**b**), the bars are colored by vertical category (red: the nominal state 13; green: stable descent; amber: sink-fast; blue: sink-slow; light grey: states never observed), and the shaded bands in panel (**a**) group the states by vertical category. The panels show that approach timesteps concentrate in the nominal configuration, with the procedurally informative remainder dominated by vertical-speed adjustments that span the single-axis deviation states.

**Figure 6:**
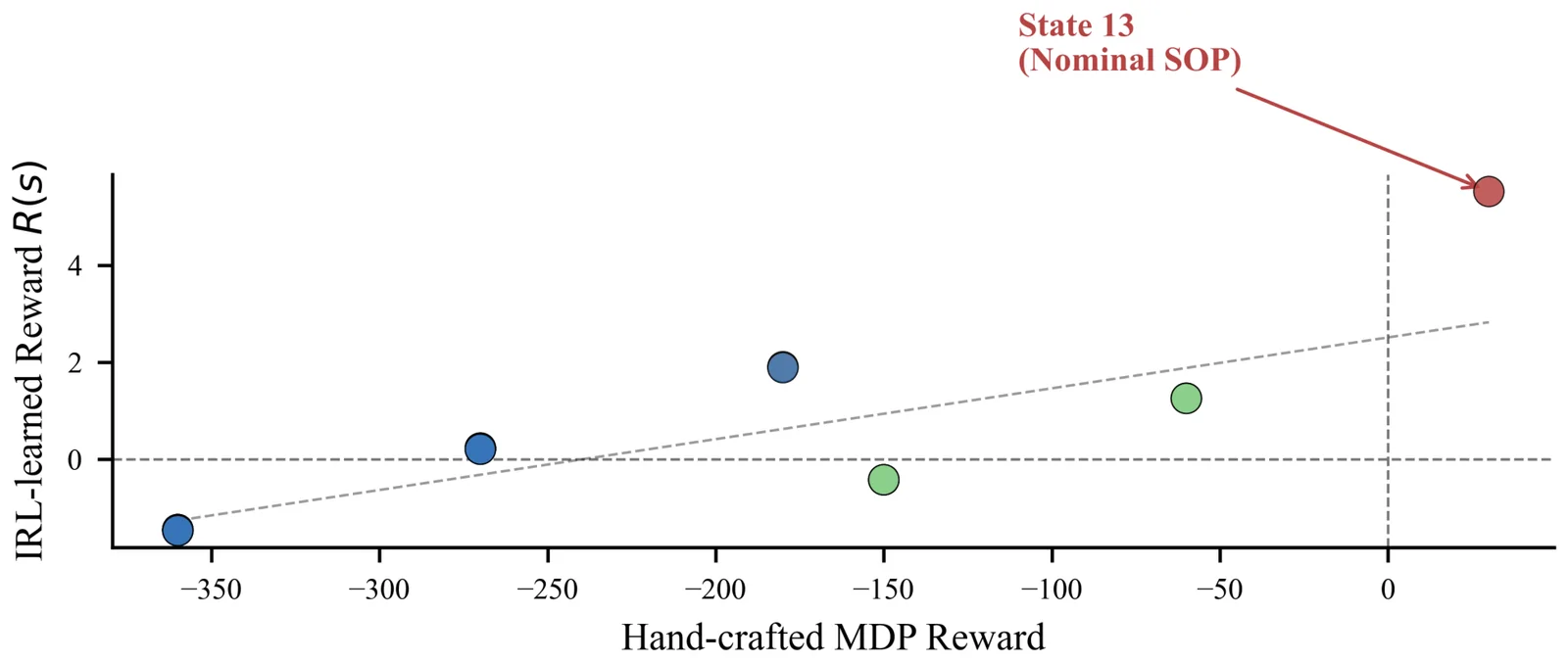
Manually defined versus IRL-learned reward function across all 27 SOP states. Each point is one SOP state, colored by its vertical category (red: the nominal state 13; green: stable descent; amber: sink-fast; blue: sink-slow); the grey dashed lines mark zero reward on each axis and the black dashed line is the fitted linear trend. The manually defined reward achieves AUROC 0.685; the IRL-learned reward achieves 0.928.

**Figure 7:**
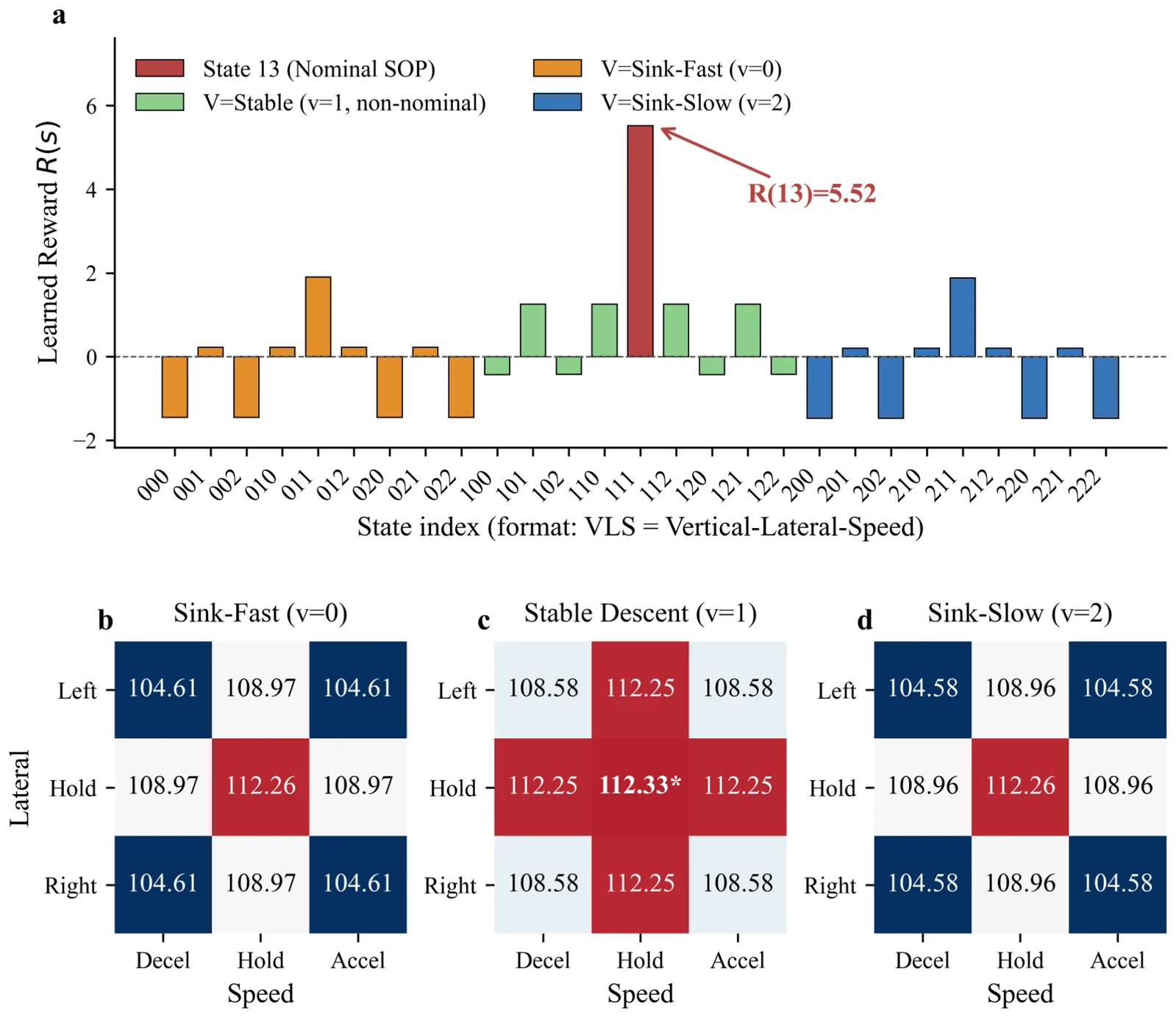
Reward function learned by IRL and soft value function. (**a**) Learned reward *R*(*s*) for all 27 SOP states. (**b**–**d**) Soft value function V*(*s*) by dimensional decomposition: (**b**) sink-fast, (**c**) stable descent, (**d**) sink-slow vertical categories. In panels (**b**–**d**), the asterisk marks the nominal state 13. Higher reward and value correspond to execution closer to the nominal SOP configuration, providing the per-state quality score used throughout the framework.

**Figure 8:**
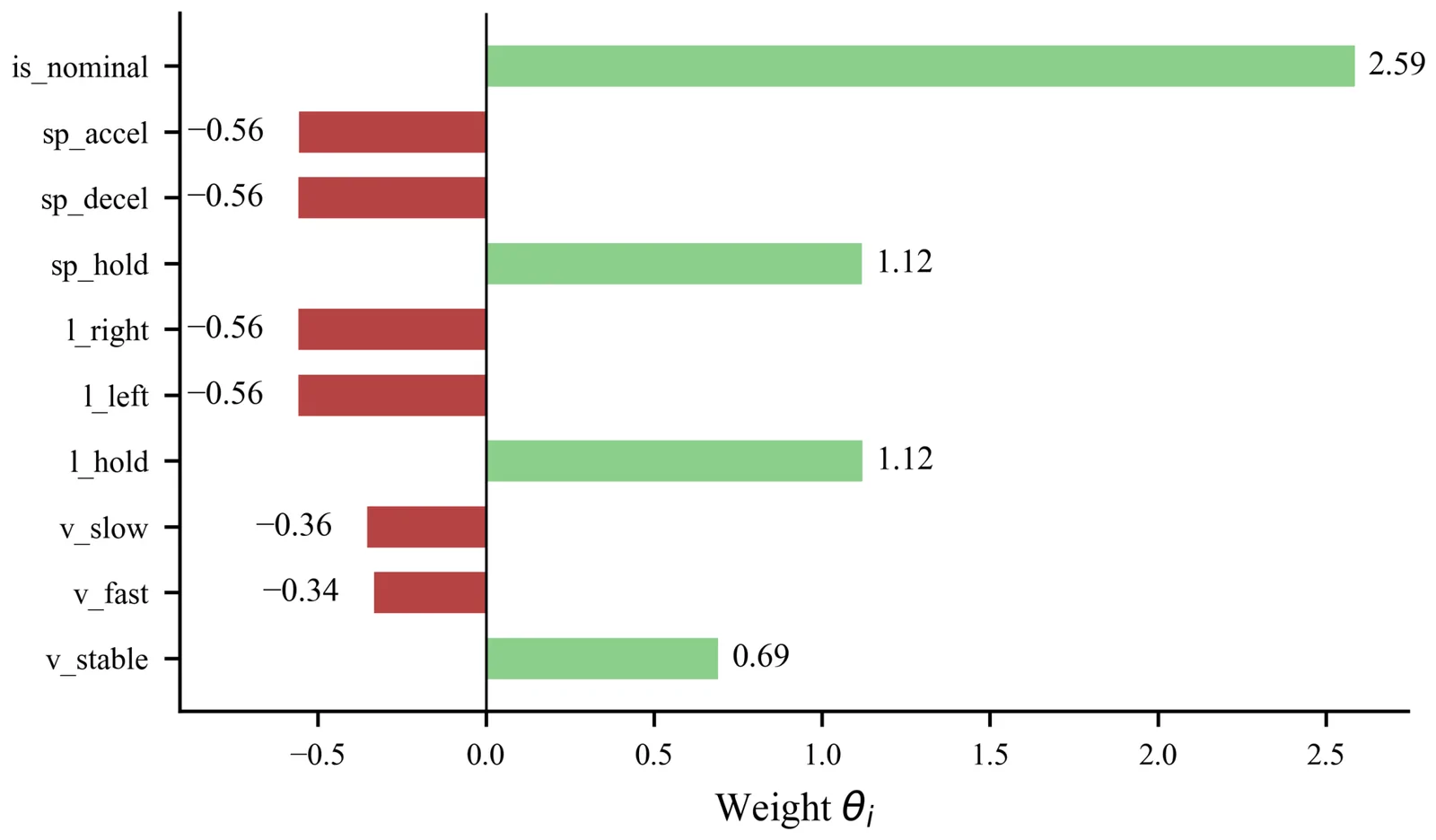
Learned feature weights θ for the linear reward function $R\left(s\right)=\theta^{⊤}\phi \left(s\right)$. Green bars denote features with positive weight and red bars features with negative weight.

**Figure 9:**
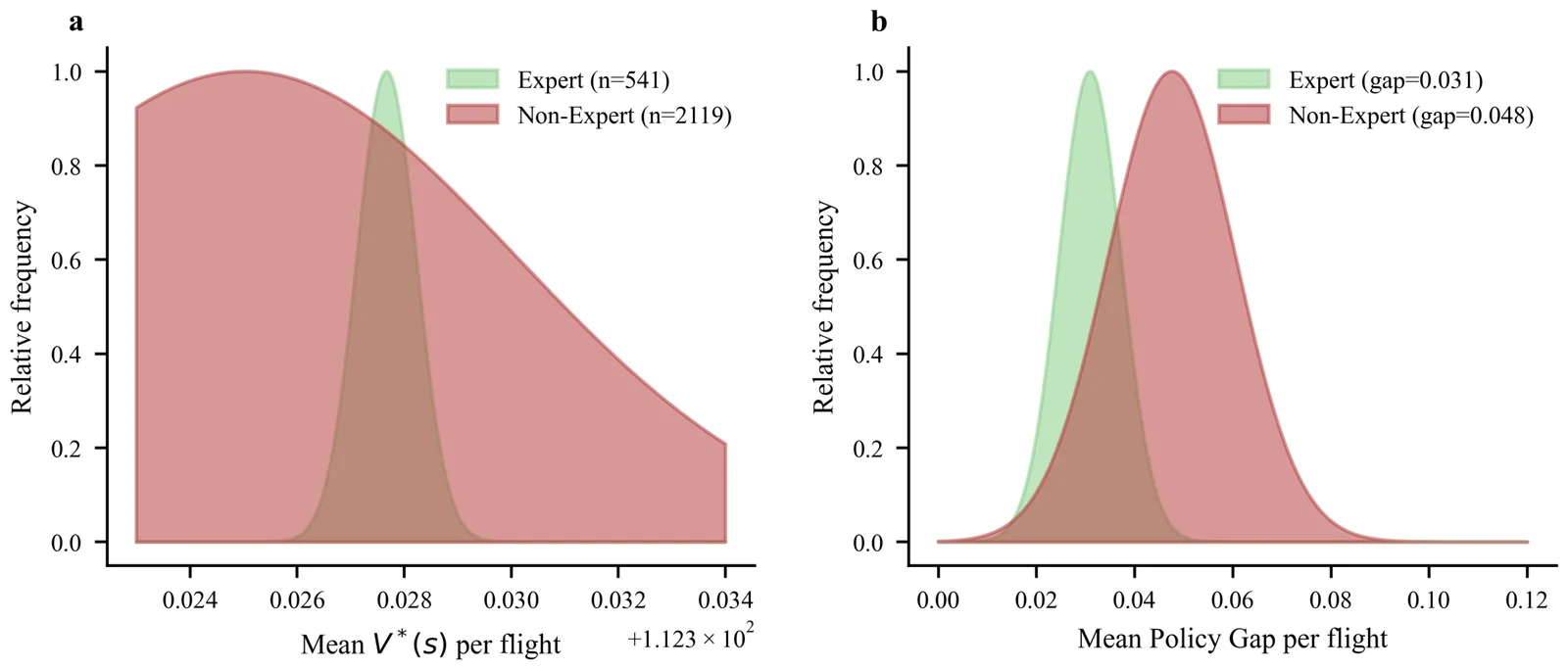
Expert versus non-expert compliance score distributions. (**a**) Value score. (**b**) Compliance gap.

**Figure 10:**
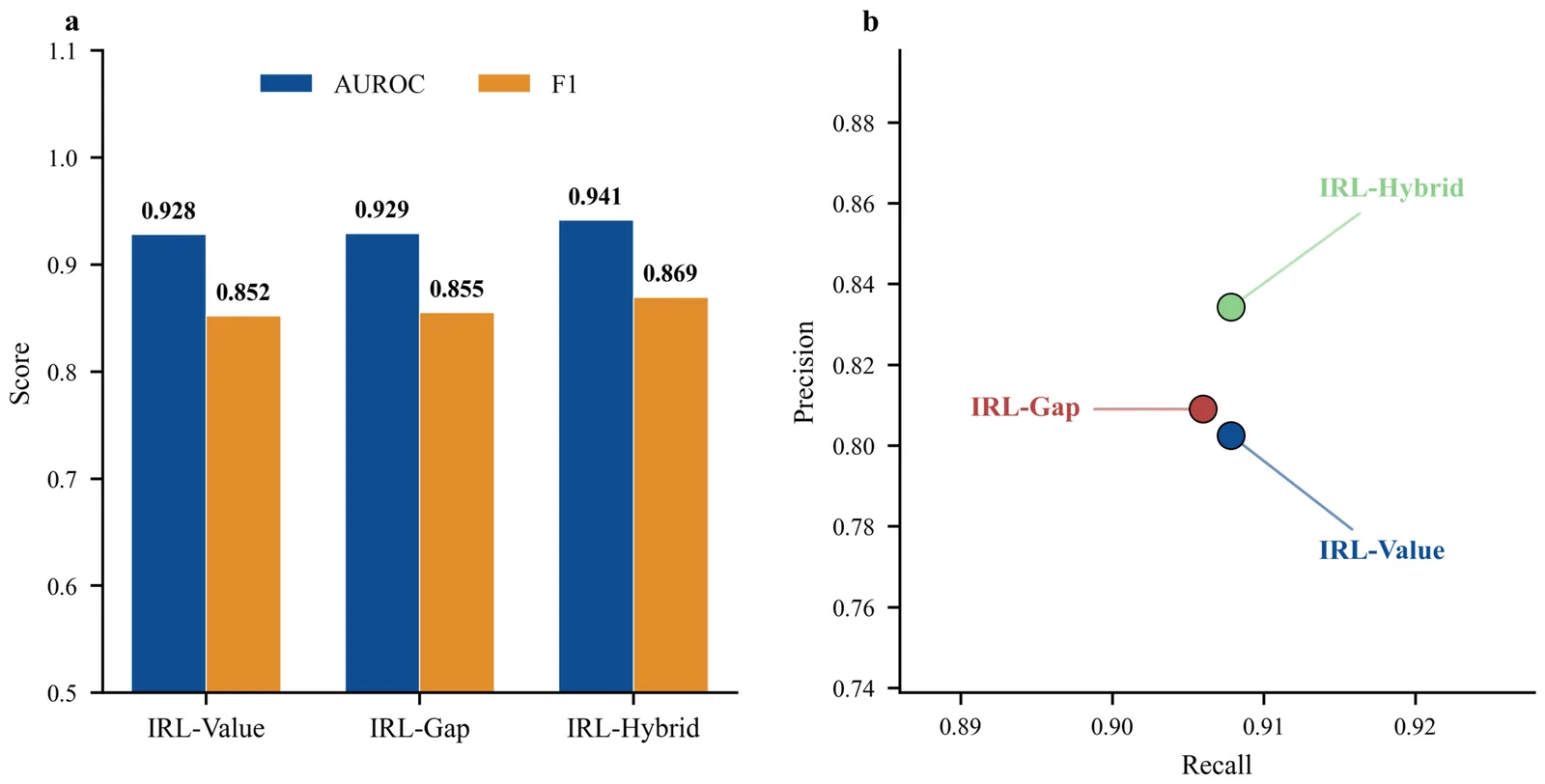
Score comparison of the three IRL-based scoring methods. (**a**) AUROC and F1 for IRL-Value, IRL-Policy Gap, and IRL-Hybrid. (**b**) Precision versus recall for the same three scores, with each point labeled by a leader line.

**Figure 11:**
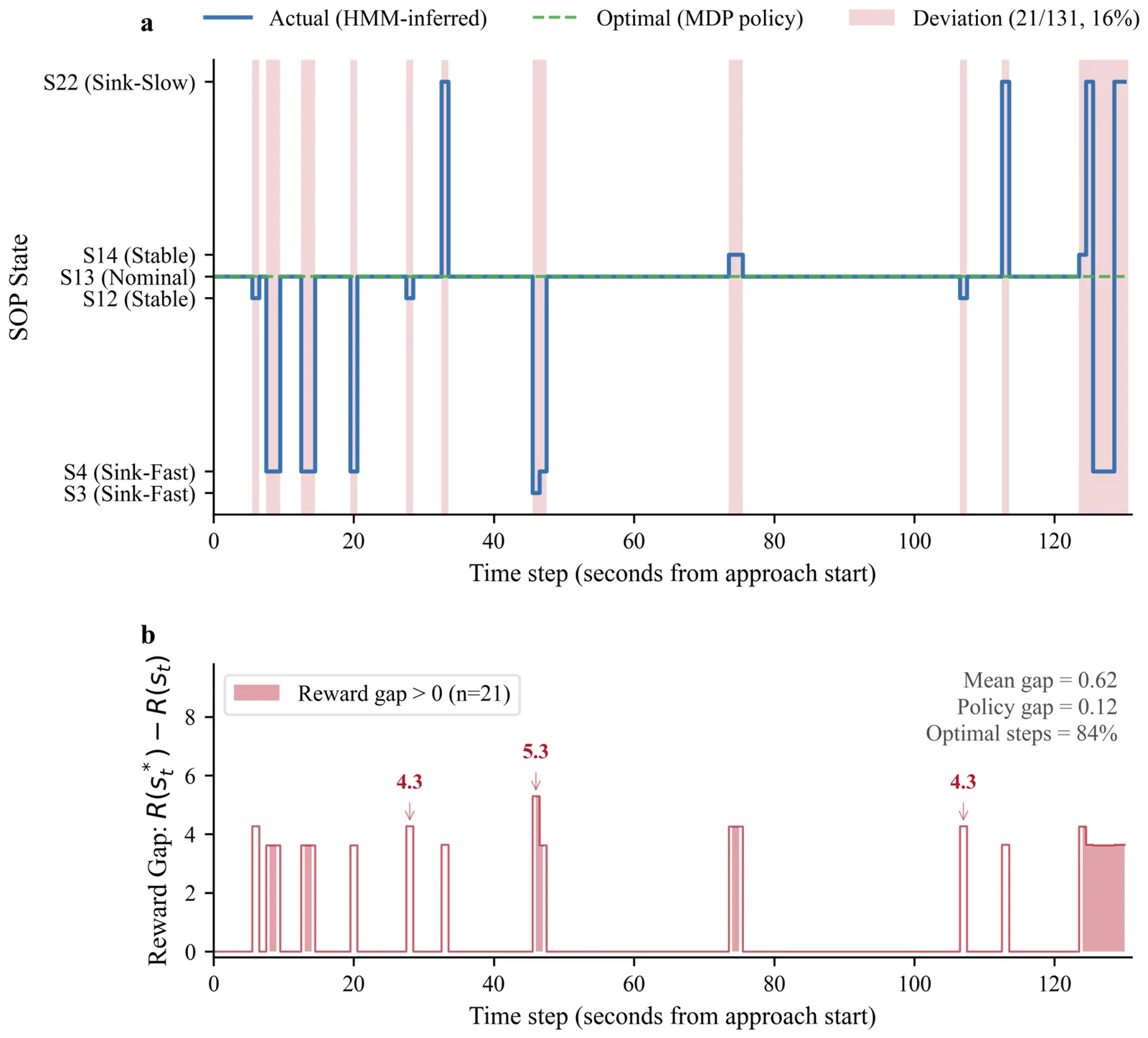
Counterfactual explanation of a representative flight. (**a**) Actual versus optimal state trajectory with deviations shaded. (**b**) Per-timestep reward gap, where the red curve traces the reward gap at each timestep and the shaded region its positive part. The shaded intervals mark the timesteps at which the inferred state departed from the SOP-optimal path and where corrective feedback would be issued.

**Figure 12:**
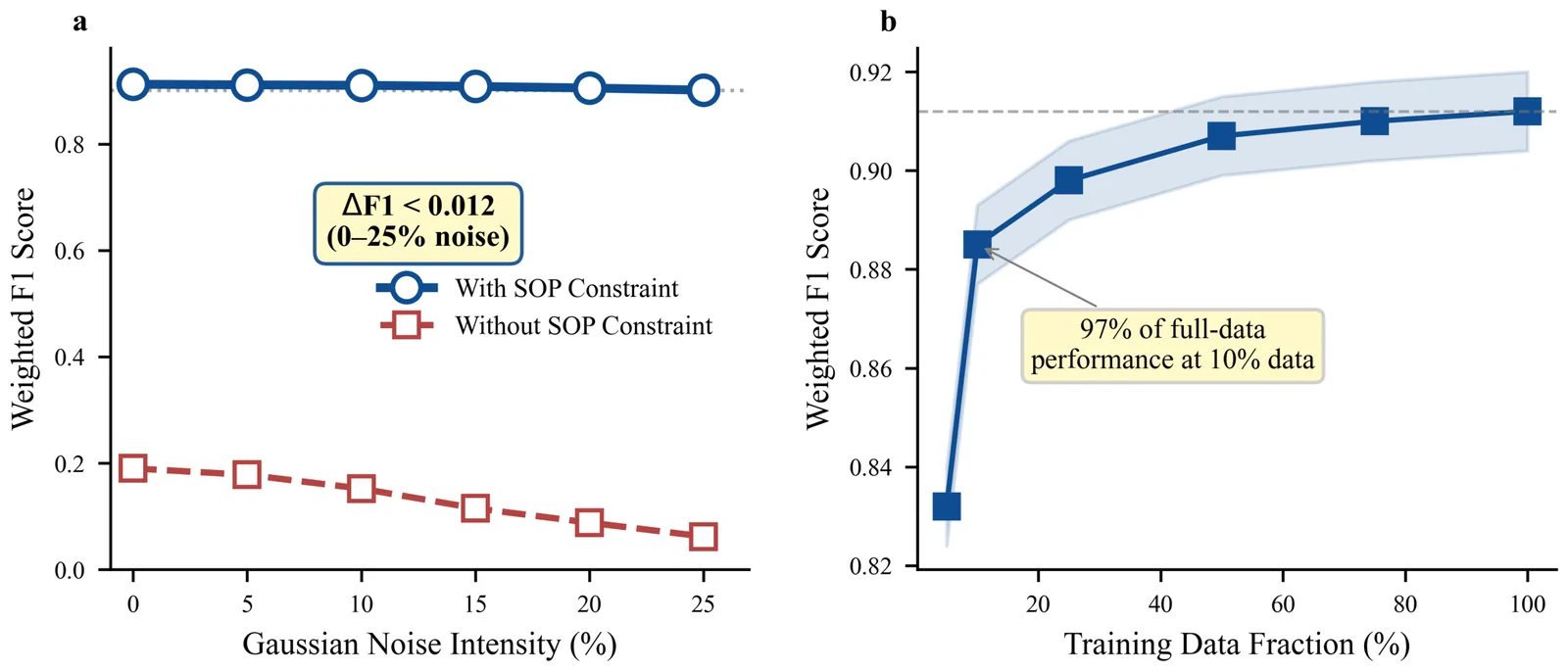
Robustness to Gaussian sensor noise and data efficiency. (**a**) Weighted F1-score under increasing Gaussian noise intensity, with and without SOP constraint. (**b**) Data efficiency: F1-score as a function of training data fraction. In panel (**a**), the red dashed curve is the variant without the SOP constraint and the grey dotted line marks F1 = 0.9; in both panels the light blue band is the uncertainty range of the corresponding solid curve and, in panel (**b**), the grey dashed line marks the full-data F1.

**Figure 13:**
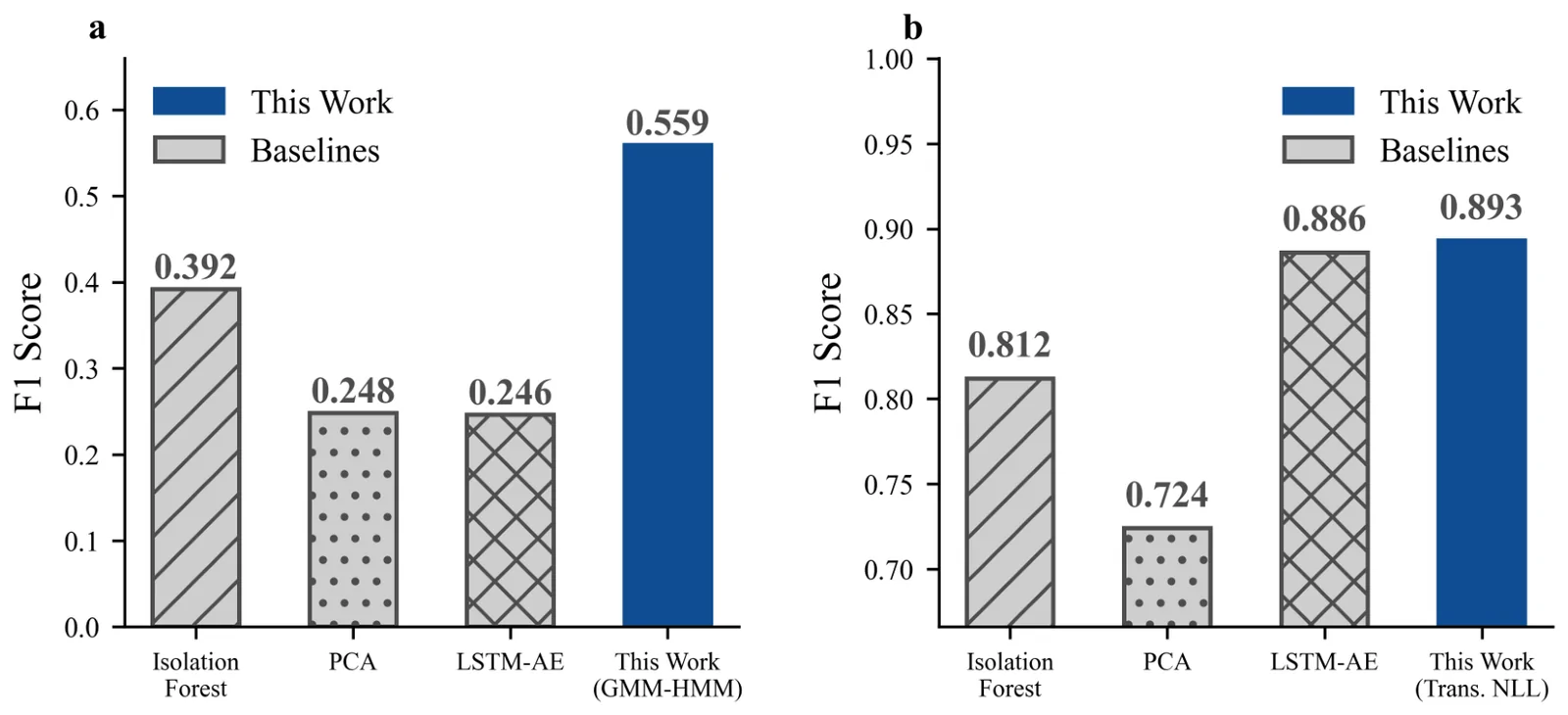
Baseline comparison. (**a**) Observation-level F1-score. (**b**) Flight-level F1-score. Bar patterns distinguish the methods (diagonal hatching: Isolation Forest; dots: PCA; cross-hatching: LSTM-AE), while the solid blue bar is the proposed framework. The proposed framework is compared with unsupervised anomaly detectors on the synthetic deviant test set; it attains competitive scores while additionally providing per-state interpretability that the baselines do not.

**Figure 14:**
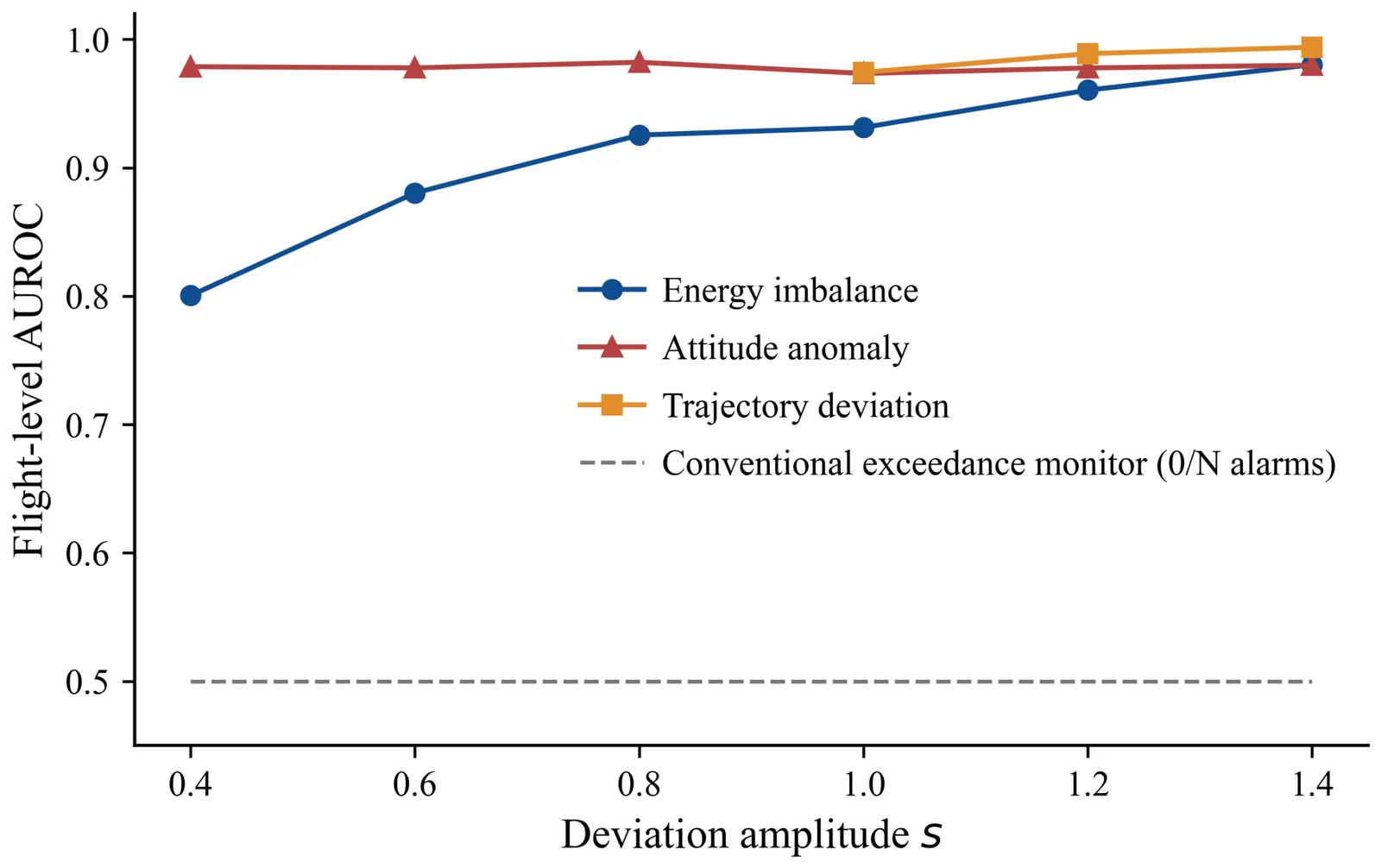
Amplitude sensitivity of the framework on fixed-severity synthetic deviations: flight-level AUROC of the IRL-hybrid score versus deviation amplitude *s* for the three deviation classes, with markers denoting the classes (circles: energy imbalance; triangles: attitude anomaly; squares: trajectory deviation). The trajectory-deviation class yields a measurable SOP-state change only for s≥1.0. The grey dashed line marks the conventional threshold-based exceedance monitor, which issues no alarm on any deviation at any amplitude because all deviations remain inside the operating envelope. The corresponding detection-latency results are reported in Table 8.

**Table 1:** Empirically active SOP states (at least 10 timesteps), ordered by descending frequency.

State	(v,l,sp)	Operational Description	Samples	%
13	(1,1,1)	Stable descent, heading hold, speed hold (nominal)	143,138	90.24%
4	(0,1,1)	Sink-fast descent, heading hold, speed hold	6060	3.82%
22	(2,1,1)	Sink-slow descent, heading hold, speed hold	4763	3.00%
14	(1,1,2)	Stable descent, heading hold, accelerating	2076	1.31%
10	(1,0,1)	Stable descent, slight left heading-rate, speed hold	990	0.62%
16	(1,2,1)	Stable descent, slight right heading-rate, speed hold	852	0.54%
5	(0,1,2)	Sink-fast descent, heading hold, accelerating	403	0.25%
1	(0,0,1)	Sink-fast descent, left heading-rate, speed hold	114	0.07%
12	(1,1,0)	Stable descent, heading hold, decelerating	94	0.06%
7	(0,2,1)	Sink-fast descent, right heading-rate, speed hold	75	0.05%

**Table 2:** Statistical baselines without learning compared with IRL-based scoring.

Method	AUROC	F1	Precision	Recall
IRL-Hybrid (this paper)	0.941	0.869	0.834	0.908
State entropy (no learning)	0.936	0.863	0.844	0.882
State-13 fraction (no learning)	0.926	0.845	0.794	0.904
Transition rate (no learning)	0.898	0.838	0.783	0.900
Manually defined MDP	0.685	—	—	—

**Table 3:** IRL-based compliance scoring performance for discriminating normal from synthetic deviant flights.

Method	AUROC	F1	Precision	Recall
IRL-Value (mean $V^{*}$)	0.928	0.852	0.802	0.908
IRL-Policy Gap (mean gap)	0.929	0.855	0.809	0.906
IRL-Hybrid (β=0.5)	0.941	0.869	0.834	0.908
Manually defined MDP	0.685	—	—	—
Transition NLL (previous best)	0.925	0.893	0.859	0.929

**Table 4:** Flight-level discrimination performance of different scoring methods.

Score Function	Aggregation	AUROC	F1	Precision/Recall
transition_nll	mean	0.9249	0.8927	0.859/0.929
transition_nll	p99	0.9246	0.8711	0.821/0.928
gmm_nll	p90	0.9212	0.8245	0.860/0.792
transition_nll	max	0.9177	0.9015	0.897/0.906
posterior	p90	0.9010	0.7953	0.736/0.865

**Table 5:** Structural ablation of the SOP transition constraint. All variants use identical GMM emission models; only the transition matrix differs.

Transition Matrix	Weighted F1	SOP-Invalid Trans.	Accuracy	S13 Pred.
SOP-Constrained (Baseline)	0.9121	0	0.9003	89.6%
Uniform (1/27)	0.8515	729	0.7920	78.8%
Data-Estimated (Unconstrained)	0.9123	119	0.9002	89.6%
Inertia-Biased (diag = 0.9)	0.8882	262	0.8539	84.7%

**Table 6:** Top-5 counterfactual state corrections across all test flights.

Actual → Counterfactual	Count	% of All Corrections
S04 (sink-fast) → S13 (stable)	2671	47.0%
S22 (sink-slow) → S04 (sink-fast)	1753	30.9%
S22 (sink-slow) → S13 (stable)	931	16.4%
S04 (sink-fast) → S22 (sink-slow)	82	1.4%
S14 (sink-slow, accel) → S13 (stable)	81	1.4%

**Table 7:** Cross-method comparison on flight-level and observation-level tasks.

Method	Flight F1	Obs. F1	Per-State Interpretable?
This framework (transition_nll)	0.893	0.559	Yes
LSTM-AE	0.886	0.246	No
Isolation Forest [43]	0.812	0.392	No
PCA	0.724	0.248	No

**Table 8:** Time from deviation onset to the first procedural flag of the framework, in timesteps. The first procedural flag is the first timestep at which the inferred SOP state departs the nominal configuration and is provided by the calibration-free IRL policy-gap signal; it fires on every retained deviation, giving a detection fraction of 1.00. Values are median, with interquartile range in square brackets.

Deviation Class	Amplitude *s*	*n*	Detection Fraction	Median Time to Flag [IQR]
Energy imbalance	0.8	150	1.00	11 [3, 25]
Energy imbalance	1.0	150	1.00	3 [0, 15]
Energy imbalance	1.2	150	1.00	1 [0, 15]
Attitude anomaly	0.8 to 1.2	80 to 92	1.00	0 [0, 0]
Trajectory deviation	1.0	150	1.00	2 [2, 2]
Trajectory deviation	1.2	150	1.00	1 [1, 1]

**Figure A1:**
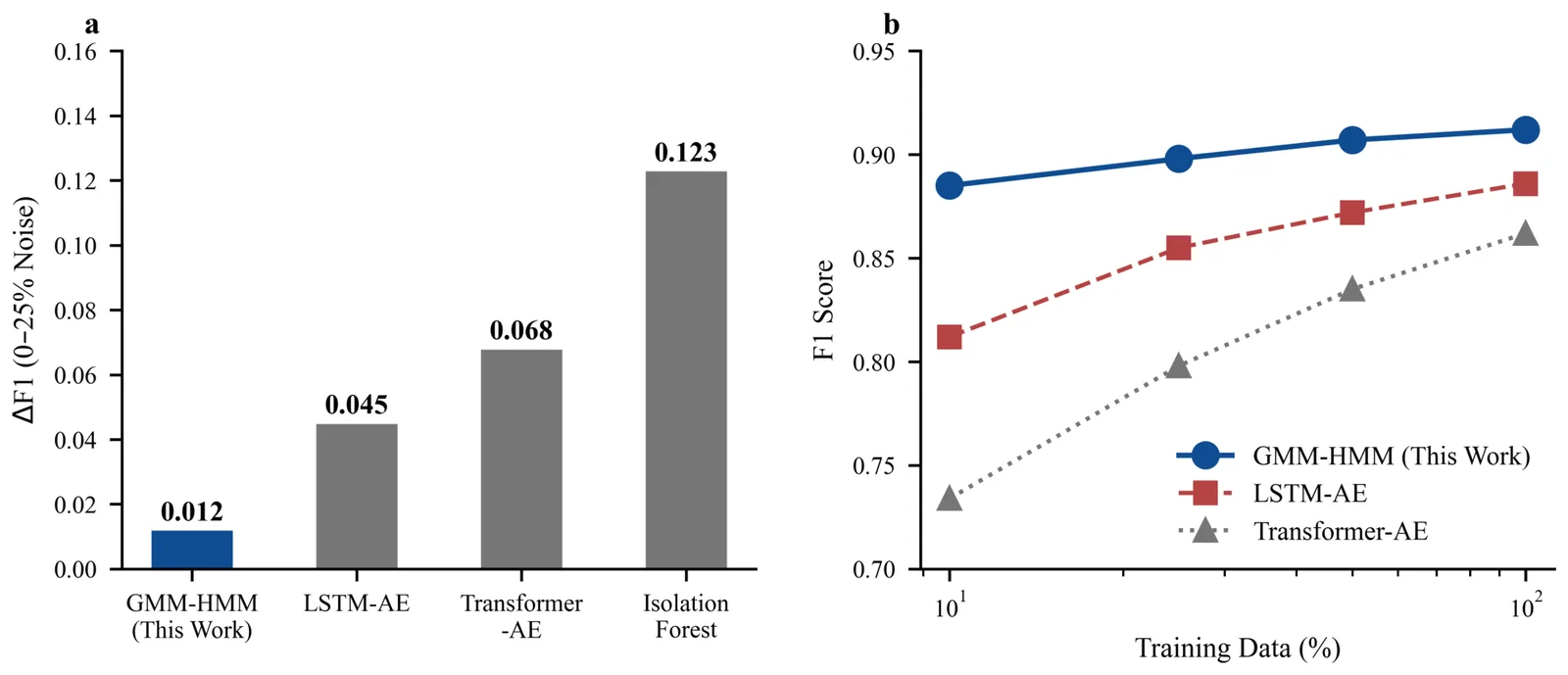
Cross-comparison of robustness and data efficiency. (**a**) Drop in F1-score from 0 to 25% Gaussian sensor noise for the proposed framework (blue) and for three baseline methods (grey). (**b**) F1-score as a function of training-data fraction on a logarithmic axis for the proposed framework, LSTM-AE, and Transformer-AE.

**Figure A2:**
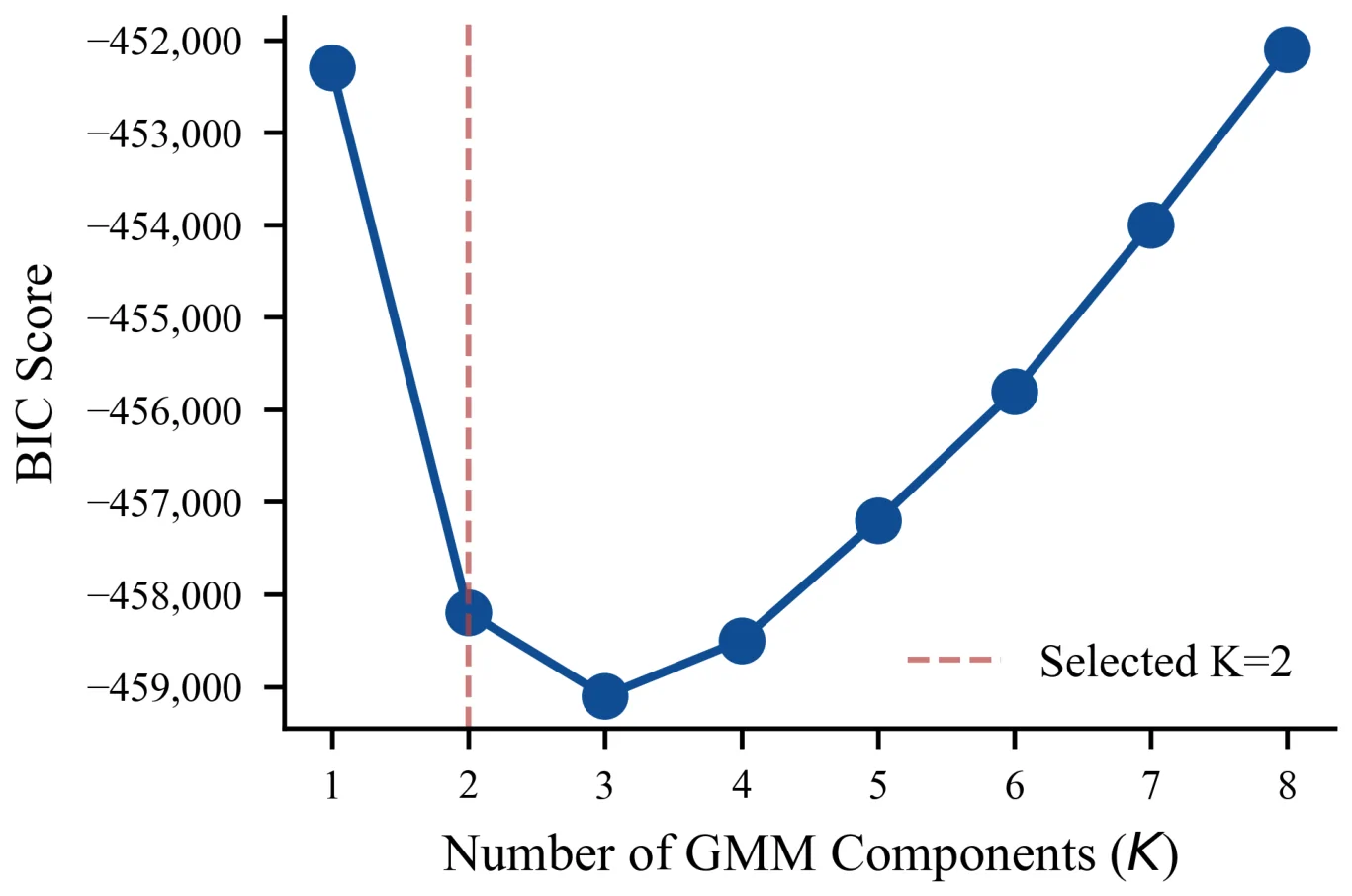
GMM component count selection via BIC. The minimum BIC at K=2 is selected, balancing aggregate inference accuracy and minority-state detection.

## Data Availability

The data presented in this study are available on request from the corresponding author. The data are not publicly available due to privacy and operational restrictions of the contributing airline operators and the national flight quality monitoring facility.

## Abbreviations

The following abbreviations are used in this manuscript:

FDM	flight data monitoring
SOP	standard operating procedure
GMM	Gaussian mixture model
HMM	hidden Markov model
IRL	inverse reinforcement learning
MDP	Markov decision process
CP	conformal prediction
QAR	quick access recorder
FOQA	flight operational quality assurance
SPI	safety performance indicator
AUROC	area under the receiver operating characteristic
FAR	false alarm rate
NLL	negative log-likelihood
EM	expectation-maximization
FCOM	flight crew operating manual
SMS	safety management system
ICAO	International Civil Aviation Organization
LSTM	long short-term memory
VI	value iteration
BIC	Bayesian information criterion
F1	harmonic mean of precision and recall
PCA	principal component analysis

## Appendix A. Supplementary Figures and Tables

This appendix contains supporting experimental results, including per-state inference metrics and a compact confusion analysis for the imbalanced state-inference task, and hyperparameter sensitivity details.

**Table A1:** Per-type detection performance for three synthetic anomaly categories.

Anomaly Type	Best Score Method	AUROC	F1
Trajectory Deviation	gmm_nll_p90	0.954	0.844
Energy Imbalance	transition_nll_mean	0.905	0.808
Attitude Anomaly	posterior_p90	0.877	0.273

**Table A2:** Summary of hyperparameter sensitivity analysis.

Sweep	Parameter	Range Tested	Stability
S1	Discount factor *γ*	[0.70, 0.97]	τ=1.0, identical top-5
S2	Learning rate η	[0.005, 0.50]	Stable in [0.02, 0.10]
S3	Iterations *T*	[10, 500]	Converges by T=40
S4	VI tolerance $\varepsilon_{\mathrm{VI}}$	[$10^{-4}$, $10^{-12}$]	τ=1.0 (all identical)
S5	Visitation horizon *H*	[50, 1000]	τ=1.0, identical top-5
S6	Random seed (initialization)	15 seeds	Mean τ=0.892, state 13 15/15
S7	γ×η interaction	3 × 5 grid	LR is bottleneck
S8	Reward normalization	–	τ=1.0 (rank-preserving)

**Table A3:** Per-state metrics of SOP state inference on the held-out test set (665 flights, 89,881 timesteps). Only states with at least one true test sample are listed, in descending order of support.

State	Support	Precision	Recall	F1
S13	83,776	0.973	0.935	0.953
S04	3161	0.099	0.161	0.122
S22	2812	0.511	0.725	0.599
S14	64	0.322	0.594	0.418
S12	46	0.377	0.565	0.452
S03	9	0.000	0.000	0.000
S05	8	0.000	0.000	0.000
S21	3	0.400	0.667	0.500
S16	2	0.000	0.000	0.000

**Table A4:** Compact confusion matrix of SOP state inference on the held-out test set, restricted to the nominal state and the most frequent non-nominal states. Rows are true states and columns are predicted states; counts are timesteps. “Other” aggregates all states beyond the five listed, comprising the additional observed non-nominal states (S03, S05, S16, S21) and the states not observed in the test partition.

	S13	S22	S04	S14	S12	Other
S13	78,307	1131	4195	69	39	35
S22	343	2038	430	1	0	0
S04	1827	818	509	6	1	0
S14	22	0	3	38	0	1
S12	8	0	11	0	26	1
Other	5	1	2	4	3	7
